# Melatonin administration provokes the activity of dendritic reticular cells in the seminal vesicle of Soay ram during the non-breeding season

**DOI:** 10.1038/s41598-020-79529-y

**Published:** 2021-01-13

**Authors:** Hanan H. Abd-Elhafeez, A. H. S. Hassan, Manal T. Hussein

**Affiliations:** Department of Anatomy, Embryology and Histology, Faculty of Veterinary Medicine, Assiut University, Assiut, 71526 Egypt

**Keywords:** Cell biology, Immunology, Molecular biology

## Abstract

Dendritic cells (DCs) are innate immune cells which engulf, process and present antigens to the naïve T-lymphocyte cells. However, little is known about the effect of melatonin on the DCs. The present study aimed to investigate the morphology and distribution of the DCs by transmission electron microscopy and Immunohistochemistry after melatonin administration. A total of 8 out of 15 adult ram was randomly selected to receive the melatonin implant and the remaining 7 animals received melatonin free implants. DCs showed positive immunoreactivity for CD117, S-100 protein and CD34. There is an obvious increase in the number of the positive immunoreactive cells to CD3, estrogen receptor alpha and progesterone in the treated groups. The expression of CD56 and MHCII in the DCs was abundant in the treated groups. The ultrastructure study revealed that melatonin exerts a stimulatory effect on the DCs which was associated with increment in the secretory activity of DCs. The secretory activity demarcated by an obvious increase in the number of mitochondria, cisternae of rER and a well-developed Golgi apparatus. The endosomal- lysosomal system was more developed in the treated groups. A rod-shaped Birbeck granule was demonstrated in the cytoplasm of the melatonin treated group. DCs were observed in a close contact to telocytes, T-Lymphocytes, nerve fibers and blood vessels. Taken together, melatonin administration elicits a stimulatory action on the DCs and macrophages through increasing the size, the number and the endosomal compartments which may correlate to increased immunity.

## Introduction

The seminal vesicle is one of the accessory genital glands that secrete a variety of biochemical constituents encompassing ions, fructose, citric acid, prostaglandins and peptides which are important for the nourishment of spermatozoa in the seminal plasma and to regulate fertility^1^. The seminal vesicle is an androgen sensitive gland and this sensitivity varies considerably between species^2,3^.

Dendritic cells (DCs) are a heterogeneous population of the mononuclear phagocyte system that plays a necessary role in the initiation of immune response^4,5^. The dendritic cells are divided into two main subtypes, the myeloid or conventional (mDCs, cDCs) and the plasmacytoid (pDCs)^5^. The subtype pDC is responsible for the initiation of anti-viral responses because it has receptors for the recognition of viral antigens^6^. Also, the cDCs reside in many tissues of the body under the normal physiological conditions and function as immunological sensors for many pathogens^7^. Dendritic cells are widely distributed in the lymphatic tissue. In addition, they are found in the non-lymphoid organs such as the skin (Langerhans cells), heart, uterine tube, lungs, cornea and the gastrointestinal tract (interstitial DC)^8–11^. Immature dendritic cells phagocytose antigens by endocytosis or macropinocytosis then process these antigens into peptides to be presented to the major histocompatibility complex class II molecule (MHC-II) and activate the naive T- lymphocyte cells^12–14^. The DCs become mature after its stimulation with the lipopolysaccharide (LPS) and cytokines^15–17^. The macrophages are professional antigen-presenting cells (APC); however, the DCs are much more potent at initiating and expanding the secondary immune responses^18^. There are several surface markers used to characterize DCs in several tissues such as CD1, CD11c, CD13, CD14, CD56, CD34, CD68, S100 and Langerin^19,20^. Unfortunately, there is no, to date, a specific marker for the DCs subtypes. Therefore, the ultrastructure approach is considered the gold standard for identifying the DCs^21,22^. The circulating melatonin is the principal hormone synthesized and secreted rhythmically by the pineal gland^23,24^, and to a lesser extent by other tissues such as the gastrointestinal tract, skin, retina, bone marrow, ovary and testis^25,26^. The melatonin secretion influenced by the environmental photoperiod. Melatonin (5-methoxy-*N*-acetyltryptamine) regulates the circadian rhythm and the seasonal changes via its nocturnal synthesis by the pineal gland. In addition, it is also involved in many pathways including antiaging, antioxidant, free radical scavenger, anticarcinogenic and immunomodulatory effects^27–29^. The immunomodulatory effect of melatonin on the immune cells is supported by the existence of G-protein-coupled membrane receptors (MT1 and MT2 receptors). Both membrane and nuclear melatonin receptors have been recognized in immune cells^30^. Melatonin induces the proliferative response of stimulated T and B- lymphocytes through the membrane receptors. However, melatonin induces the cytokine production in the mononuclear cells such as DCs and macrophages through the nuclear receptors^31^. It was demonstrated that melatonin induces IL-12 (Interleukin-12) and IL-6 secretion in monocytes which will stimulate the secretion of IL-2 to differentiate towards T-helper phenotype^32^. Melatonin enhances the immune response through stimulation of natural killer cells, antigen presentation and phagocytosis. In our previous study we investigated a significant increase in the number of dendritic cells and its phagocytic vesicles in the adrenal gland of melatonin treated groups of Soay ram^33^. Melatonin is the main regulator of reproduction especially in the photoperiodic animals as it modulates the synthesis and secretion of gonadotrophic hormones in the hypothalamus ^34^. The exogenous melatonin treatment during the non-breeding season increases the testosterone level in the blood plasma^35,36^, the sperm quality, the testicular parameters, the spermatogenesis and fertility of rams during the long day’s periods^37,38^.

The present study was an extension from our previous study which demonstrated the stimulatory effect of melatonin on the seminal gland of Soay ram during the non-breeding season. It was proved that melatonin administration resulted in cytological signs of increase in the secretory activity and height of the principal cells lining the glandular epithelium of the gland. In addition, we investigated a significant increase in the number of telocytes and its stromal synapses with immune cells such as macrophages and mast cells after melatonin administration^39,40^. Therefore, the current study continues our work to understand the broader effect of melatonin on the immunity and validate its use in practice as an enhancer for immunity and fertility. To our knowledge there has been no study about the effect of melatonin on the dendritic cells biology. Therefore, the objective of the present study was to characterize the morphology and the distribution of the dendritic cells in the seminal vesicle of Soay ram after melatonin administration using the transmission electron microscopy and the immunohistochemical study.

## Materials and methods

### Animals and experimental design

Experiments were conducted in accordance with the U.K. Animals (Scientific Procedures) Act of 1986 in MRC Reproductive Biology Unit, Centre for reproductive biology, Edinburgh, Scotland, U.K. This study was performed in strict accordance with the relevant guidelines and ethical regulations (experiment No. S/17353). The animals and the experimental design have been discussed before by^39,41^. In brief, the animals of Soay Ram breed (*Ovis aries*) were obtained from specialist breeders in Scotland. The seminal glands of 15 adult Soay rams (aged 1.5 years) were used in this study. At the end of May, eight animals were given a subcutaneous implant containing melatonin (treated group), while another group of seven animals were given empty implants (control group). The melatonin implants were made of Silastic sheeting (500-1DOw Corning sheeting, Midland, MI, USA) sealed into an envelope, containing 1.0 gm melatonin (Sigma Chemical, Poole, Dorset, UK). The implants were placed subcutaneously above the rib cage using local anesthesia and left throughout the time of experiment. Such implants have been previously shown to maintain a constant supply of 200–500 pg melatonin**/**ml plasma for many months without interfering with the endogenous supply of the secreted melatonin^42^. Eleven weeks after the onset of the experiment, all rams were sacrificed by intravenous barbiturate injection, the seminal glands were carefully excised, and small samples were processed for light and electron microscopic examination. Both light and electron microscopic examinations were done at Assiut University, Egypt.

#### Light microscopy

The samples were obtained from the seminal glands of both control and melatonin treated groups, dissected and immediately fixed in 4% paraformaldehyde in 0.1 M phosphate buffered saline (PBS, pH 7.4) over night (ON) at 4 °C. The fixed samples were dehydrated in an ascending series of ethanol, cleared in methyl benzoate then embedded in paraffin wax. Samples were cut at 5–8 μm in thickness transversely and longitudinally. The staining protocols and used procedures were carried out following the descriptions of the histological techniques as reported by^43^. The sections were stained with Haematoxylin and Eosin for demonstrating the general histological structure of the examined seminal gland and to identify the DCs.

#### Semi-thin sections and transmission electron microscopic studies

The procedure of staining was done according to^44,45^. The seminal glands were preserved by immersion in a mixture of 5% paraformaldehyde–glutaraldehyde fixative overnight^46^. After fixation, the samples were washed in phosphate buffer solution and osmicated with 1% osmium tetroxide in 0.1 mol/L Na-phosphate buffer at pH 7.3. After that, the samples were dehydrated in series of ethanol followed by propylene oxide and embedded in Araldite. Semi-thin sections were cut at 1 µm thickness with a Reichert Ultracut (Leica, Germany) and stained with toluidine blue for light microscopy.

Ultrathin sections were done with Ultrotom VRV (LKB Bromma, Germany). The sections (70 nm) were stained with uranyl acetate and lead citrate^47^ and examined in a JEOL 100CX II transmission electron microscope (TEM) (JEOL, Tokyo, Japan) at the Electron Microscopy Unit of Assiut University.

#### Digitally colorization of TEM images

To increase the visual contrast between several structures on the same electron micrograph, we have digitally colored specific elements [e.g., dendritic cells (DCs), telocytes (TCs), nerve fibers and lymphocytes] to make them more visible. All the elements were carefully hand colored in Adobe Photoshop software version 6. The methods were used by^41,48–51^.

#### CMEIAS color segmentation (for the supplementary images)

Negative images were obtained using the CMEIAS color segmentation software, which processes color images by segmenting the object of interest in the foreground from the background^52^. This process is conducted by the following steps: open image file with CMEIAS color segmentation, then select “Process” from the menu items and subsequently choose “Negative image”^53–55^.

#### Immunohistochemistry technique

To demonstrate the dendritic cells in the seminal glands of Soay rams, six different antibodies were used including, anti-estrogen receptor alpha (ER-α), anti-progesterone, CD3, CD117, CD34, MHCII, CD56 and S-100 protein (Table 1). These antibodies have been validated in various studies in sheep^33,40,56^. In addition, most of used antibodies in the current study their cross reactivity was proved in bovine and goat (ruminants) according to manufacturer data sheet or not tested yet.

**Table 1:** Identity, sources, and working dilutions of primary and secondary antibodies.

Target	Primary antibody suppliers	Primary antibody origin (catalog no.)	Primary antibody dilution	Primary antibody incubation period	Biotinylated secondary antibody
S-100 protein	Genemed Biotechnologies	Rabbit (pc; 61-0061)	1:200	1 h at RT	Anti-mouse IgG + anti-rabbit IgG (biotinylated goat anti-polyvalent)^b^
Estrogen receptor (SP1)	Genemed Biotechnologies	Rabbit (pc; RM_9101-S0	1:200	1 h at RT	Anti-mouse IgG + anti-rabbit IgG (biotinylated goat anti-polyvalent)^b^
Progesterone receptor	Immunotech	Mouse (mc; PR10A9)	1:50	Overnight at 4 °C	(Rabbit anti-mouse IgG)^b^
CD 34 (clone QBE d/10)	ThermoFisher Scientific	Mouse (mc; MS-363-R7)	Ready to use	1 h at RT	Anti-mouse IgG + anti-rabbit IgG (biotinylated goat anti-polyvalent)^a^
anti-c-kit (CD117)	Genemed Biotechnologies	Rabbit (pc)	1:50	1 h at RT	Anti-mouse IgG + anti-rabbit IgG (biotinylated goat anti-polyvalent)^a^
CD3	Novus Biologicals	Rabbit (pc, NB 100-2000)	1: 100	Overnight at 4 °C	(goat anti-rabbit IgG)^b^
CD56	Santa Cruz biotech	Goat (pc, Sc 1507)	1:100	Overnight at 4 °C	(Donkey anti-goat IgG)^b^
MHCII	Abcam	Mouse (mc, ab23990)	1:100	Overnight at 4 °C	(Donkey anti-mouse IgG)^b^

*pc* polyclonal, *mc* monoclonal, *RT *room temperature.

^a^From ThermoFisher Scientific/Lab Vision, Fremont, CA, USA.

^b^From Dako, Hamburg, Germany.

Immunohistochemistry staining technique had been performed on the paraffin fixed tissue. Sections were deparaffinized with xylene and hydrated with a descending grade of ethanol then washed with 0.1 M PBS (3 × 10 min). To inhibit the endogenous peroxidase activity the sections was incubated with 3% H_2_O_2_ in H_2_O for 20 min at the room temperature (RT), followed by intense washing under running tap water for an additional 10 min. To decrease the masking of antigen epitopes, the antigen retrieval was carried out using 0.1 M sodium citrate buffer solution (pH 6) for 15 min using a microwave (750 Watt). Then, sections were cooled to room temperature for 20 min and washed with PBS (pH 7.4, for 10 min). The sections were covered with Ultra V Block (ThermoFisher Scientific, TP-015-UB) for 5 min at room temperature to minimize the non-specific antibody binding. Then, the sections were incubated with the primary antibodies diluted in the blocking solution [1.5% normal donkey serum (NDS) + 0.2% Triton-X 100/PBS] (Table 1). The sections were rinsed in 0.2% Triton-X 100/PBS and followed by incubation with a biotinylated secondary antibody (Table 1) for 1 h at RT. Then, sections were washed by PBS (pH 7.4, 3 times for 5 minutes) and, subsequently incubated with streptavidin-peroxidase complex (ThermoFisher Scientific, TS-015-HR) for 15 min at room temperature. Visualization of the reaction was carried out with a drop of DAB plus chromogen (ThermoFisher Scientific, TA-001-HCX) to 2 ml of DAB plus substrate (ThermoFisher Scientific, TP-015-HSX) which applied on the sections for 5–10 min. The sections were counterstained with Harris haematoxylin for 30 s. The sections were dehydrated in a graded series of ethanol, cleared with xylene and covered with DPX. Immunohistochemical staining was evaluated by LeitzDialux 20 Microscope and photos were photographed by cannon digital camera (Cannon Powershot A95). IHC staining was quantified within control and melatonin treated groups for ER-α, CD3 and Progesterone.

#### Morphometrical and statistical analysis

The morphometric studies were performed on both light and electron- microscopic images of the seminal glands of both control and treated animals using Image-J software. The measurements were carried out on 15 randomly selected sections of each gland per animal (5 different areas were measured from each section) as follows: the interstitial connective tissue/glandular tissue ratio per 20 mm^2^ using 40× objective, the cross sectional area of dendritic cells in the glandular epithelium and the surrounding interstitium per 20 μm^2^ in TEM images. The number of dendritic cells in the glandular epithelium of histological sections, semi-thin sections and CD3, estrogen receptor alpha (ER-α) and progesterone immunostained sections were performed per 20 mm^2^ using 20× objective. Per each animal the quantification were performed from each fifth section (to avoid double counting of the same cells). All the data were expressed as mean± SE (standard error) which was statistically analyzed using “T-Test Graphpad prism Software” (Version 6.05, International Scientific Community) to compare between different measurements of both control and treated animals. Differences were considered significant if P <0.05 (*) and highly significant if P < 0.01 (**).

### Ethical approval

"All methods were performed in accordance with the relevant guidelines and regulations" Experiments No. (S/17353) were conducted in accordance with the U.K. Animals (Scientific Procedures) Act of 1986 in MRC Reproductive Biology Unit, Centre for reproductive biology, Edinburgh, Scotland, U.K. This study was carried out in strict accordance with the national and ethical regulations and care of animals was in accordance with institutional guidelines. The protocol was approved by the Central Office for Research Ethics Committees (COREC) in Eastarbourne Terrace, United Kingdom.

## Results

### General morphology (characterization) of the dendritic cells

#### Histological analysis

**Figure 1:**
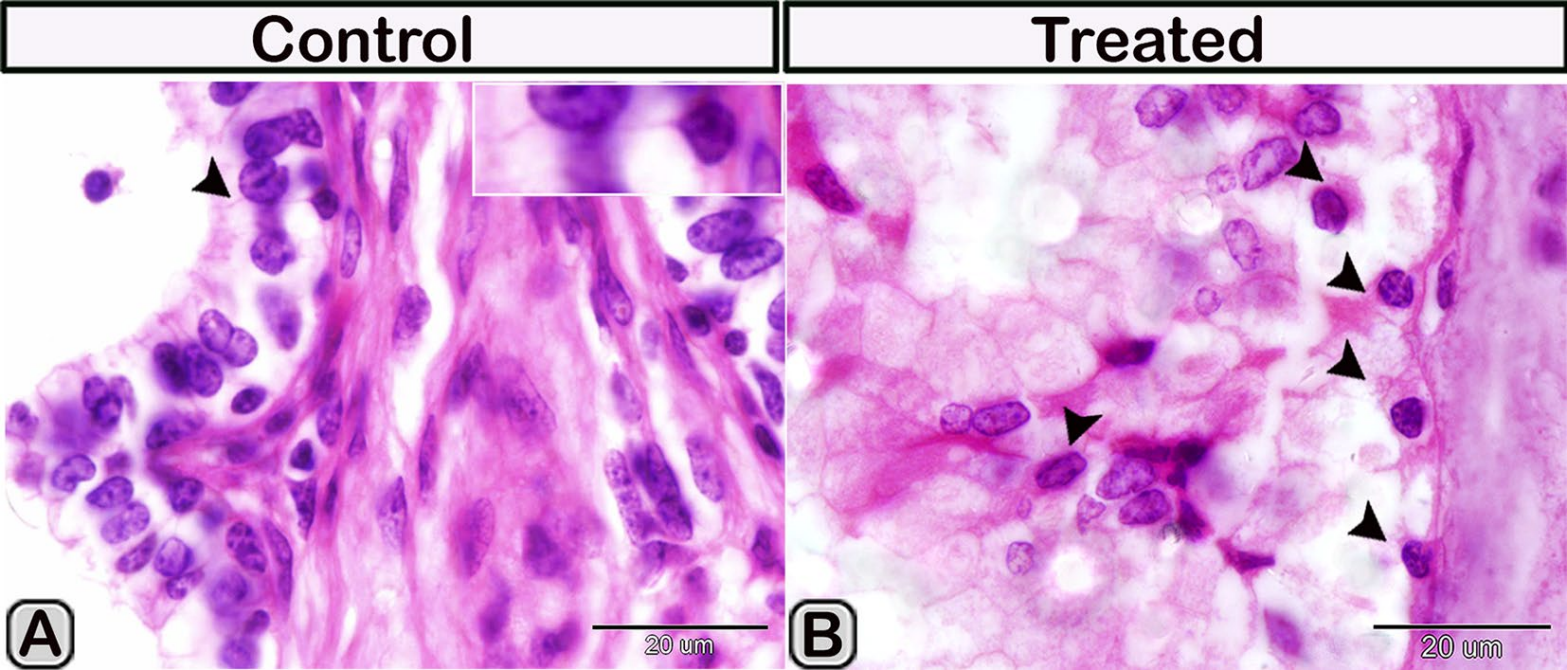
Paraffin sections stained with H&E showing the dendritic cells residing among the glandular epithelium lining the seminal gland of the Soay ram. **(A)** DCs were small with a deeply stained nucleus, acidophilic cytoplasm and a cytoplasmic process directed toward the lumen of the gland (arrowhead). **(B)** The number of DCs was obviously increased in melatonin treated groups (arrowhead).

**Figure 2:**
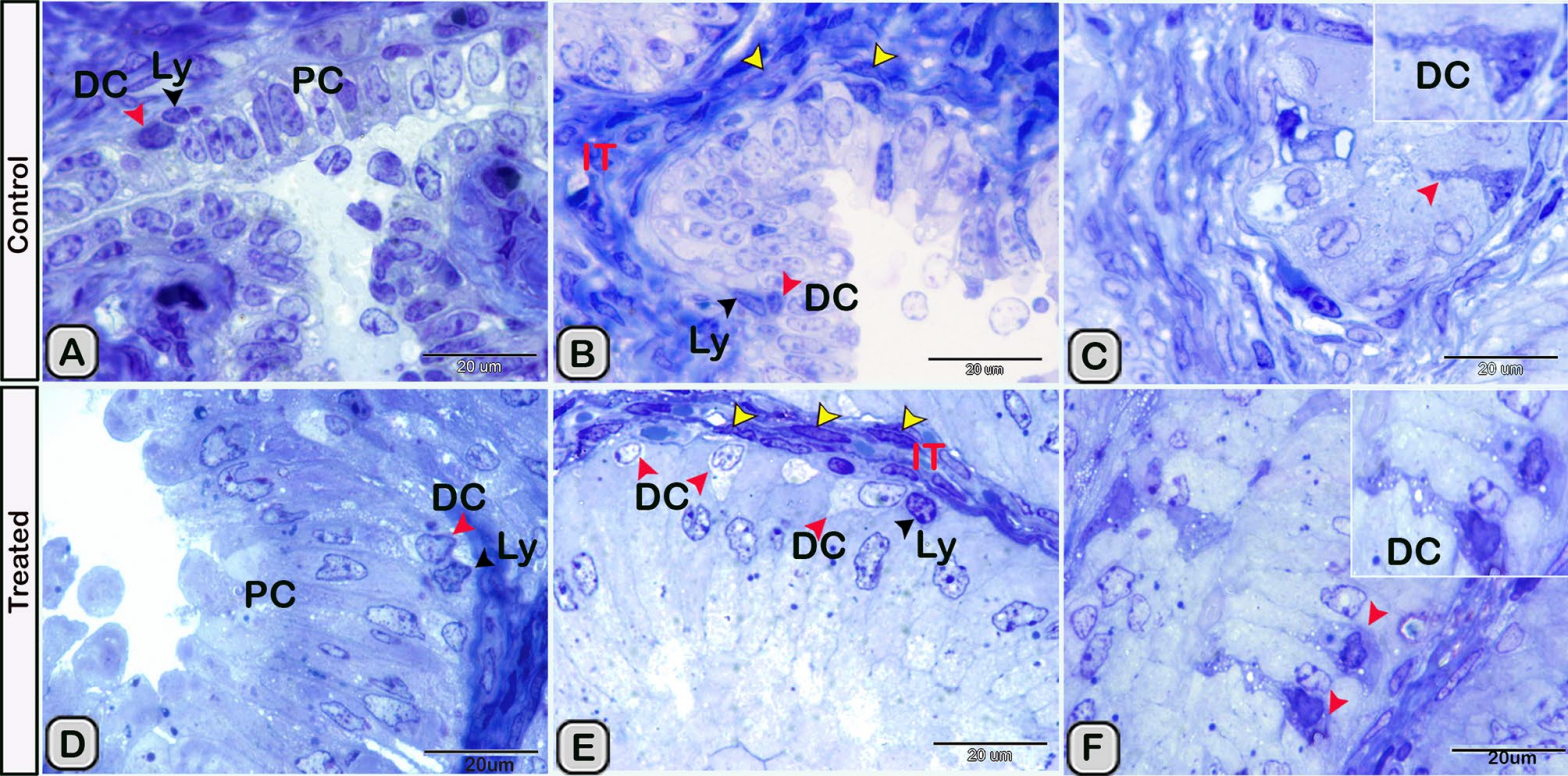
**(A,B)** Semi-thin sections stained with Toluidine blue showing the DCs (red arrowheads) in the control groups near the basement membrane lining the glandular epithelium and its close association to T- lymphocytes (black arrowhead) and telocytes (TCs, yellow arrowheads) in the interstitium (IT). **(C)** A triangular-shaped dendritic cell with a few vesicles in the cytoplasm and a long cytoplasmic process directed toward the lumen of the gland was observed. **(D,E)** In the melatonin treated groups, DCs (red arrowheads) were closely associated to T-lymphocytes (Ly, black arrowheads) and telocytes (yellow arrowheads) in the interstitium (IT). DCs were larger in size and their nucleus was euchromatic and more indented in comparison to control groups. Notice, the epithelium of the principal cells (PC) is higher in melatonin groups compared to control ones. **(F)** The cytoplasm of dendritic cells had large amount of the secretory vesicles in comparison to the control groups.

**Figure 3:**
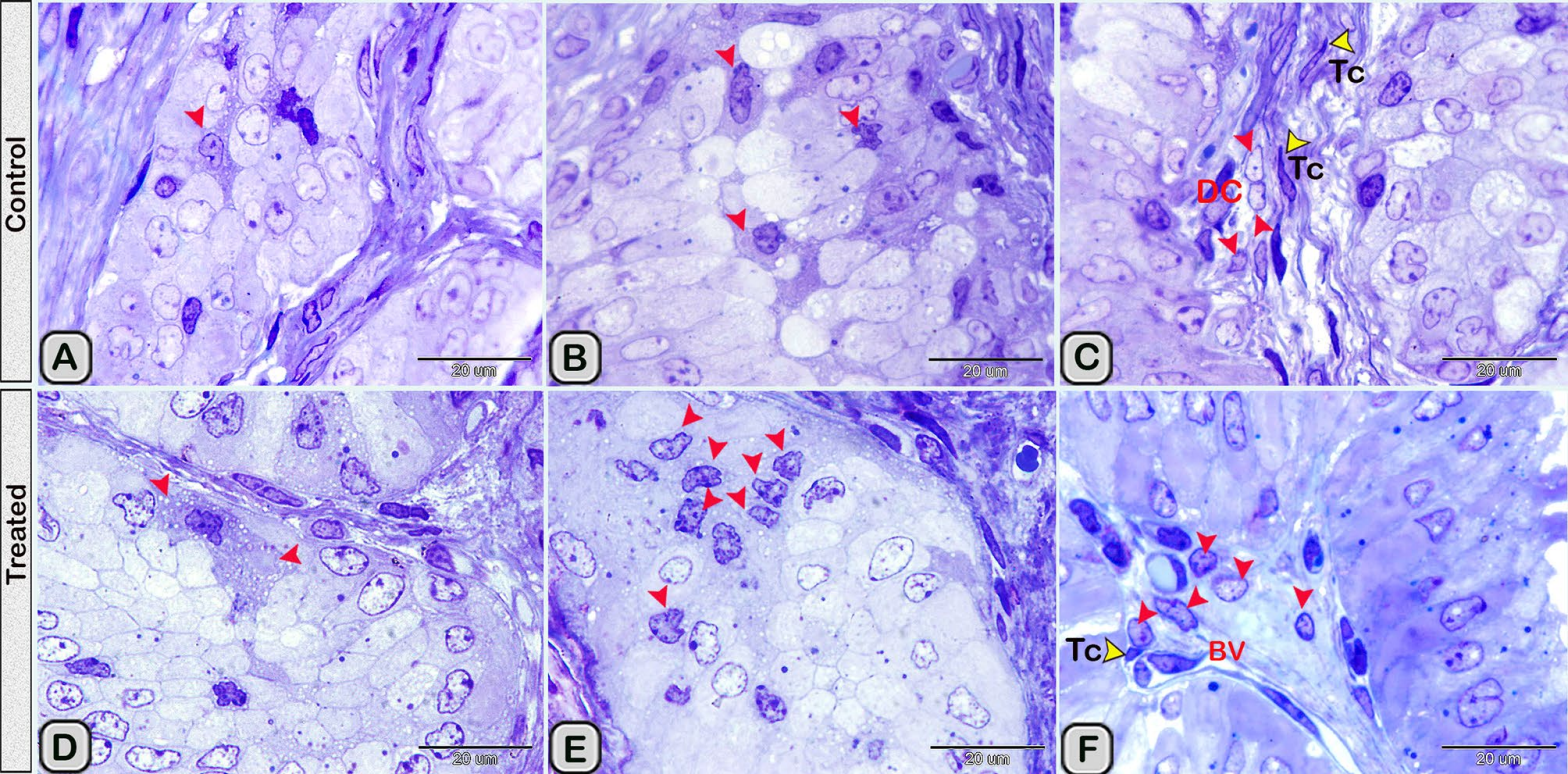
**(A,B)** The free movable DCs (red arrowheads) were observed within the glandular epithelium lining the seminal gland of the control groups. **(C)** Interstitial DCs (red arrowheads) were observed in a close contact to telocytes (TC, yellow arrowheads). (**D–F**) Semi-thin sections stained with Toluidine blue in the melatonin treated groups. **(D,E)** free movable dendritic cells were larger in size and more abundant in comparison to the control ones (red arrowheads). **(F)** Interstitial DCs (red arrowheads) were more abundant compared to the control group and in a close contact to telocytes (TC) and blood vessels (BVs).

In the paraffin sections stained with H&E of the control groups, the dendritic cells were demonstrated residing among the glandular epithelium lining the seminal vesicle. The cells were small with a deeply stained nucleus, acidophilic cytoplasm and a cytoplasmic process directed toward the lumen of the gland (Fig. 1A). However, in the melatonin treated groups, the number of dendritic cells in the epithelial lining the gland was obviously increased in comparison to the control ones (Fig. 1B, Table 2). In Toluidine blue stained semi-thin sections of the control groups, DCs were observed near the basement membrane lining the glandular epithelium and in a close association to T- lymphocytes and telocytes (TCs) in the interstitium (Fig. 2A,B). Lymphocytes are usually smaller in size than DCs. Lymphocytes have a small spherical or oval nucleus and abundant darkly stained chromatin. A triangular-shaped dendritic cell with a few vesicles in the cytoplasm and a long cytoplasmic process directed toward the lumen of the gland was observed (Fig. 2C). Whereas, in the melatonin treated groups, DCs were larger in size and their nucleus was euchromatic and more indented in comparison to control groups. The associated T-lymphocytes were active, larger in size and their nucleus was vesicular with chess like appearance of chromatin (Fig. 2D,E). The cytoplasm of dendritic cells had large amount of the secretory vesicles in comparison to the control groups (Fig. 2F). The free movable DCs were investigated within the glandular epithelium of the seminal gland (Fig. 3A,B). The interstitial DCs were investigated in the interstitium of the gland and were in close contact with telocytes (Fig. 3C). The movable DCs were larger in size and more abundant in the melatonin treated groups when compared to the control (Fig. 3D,E and Table 2). In addition, the interstitial DCs were more abundant and were in close contact with telocytes and blood vessels (Fig. 3F).

**Table 2:** The morphometric measurements of the control and melatonin treated groups.

Measurements	Control	After melatonin treatment
Ratio of interstitial tissue/glandular tissue (Fig. 2 and other serial semithin sections)	0.494 ± 0.019	0.241 ± 0.021*
Cross sectional area of dendritic cells in the glandular epithelium (TEM images , Figs. 10, 12, 13, 14)	20.97 ± 4.155	48.72 ± 7.528*
Cross sectional area of dendritic cells in the interstitium (TEM images, Figs. 10, 16)	17.99 ± 3.972	15.78 ± 3.219 (ns)
Number of DCs in the glandular epithelium of the histological and semi-thin sections (Figs. 1, 2, 3)	3.667 ± 0.2562	8.917 ± 0.8569**
Number of DCs in the glandular epithelium of estrogen alpha receptors (Fig. 6)	3.571 ± 0.4809	6.543 ± 0.5936***
Number of DCs in the glandular epithelium of progesterone receptors (Fig. 7)	3.000 ± 0.2582	6.500 ± 0.5627***
Number of DCs in the glandular epithelium of CD3 (Fig. 5)	4.750 ± 0.4787	8.500 ± 0.6455**

The measurements are expressed as the mean ± SE.

When P < 0.05 (*) is significant, P < 0.01 (**) and P < 0.01 (***) is highly significant.

#### Immunohistochemical analysis

**Figure 4:**
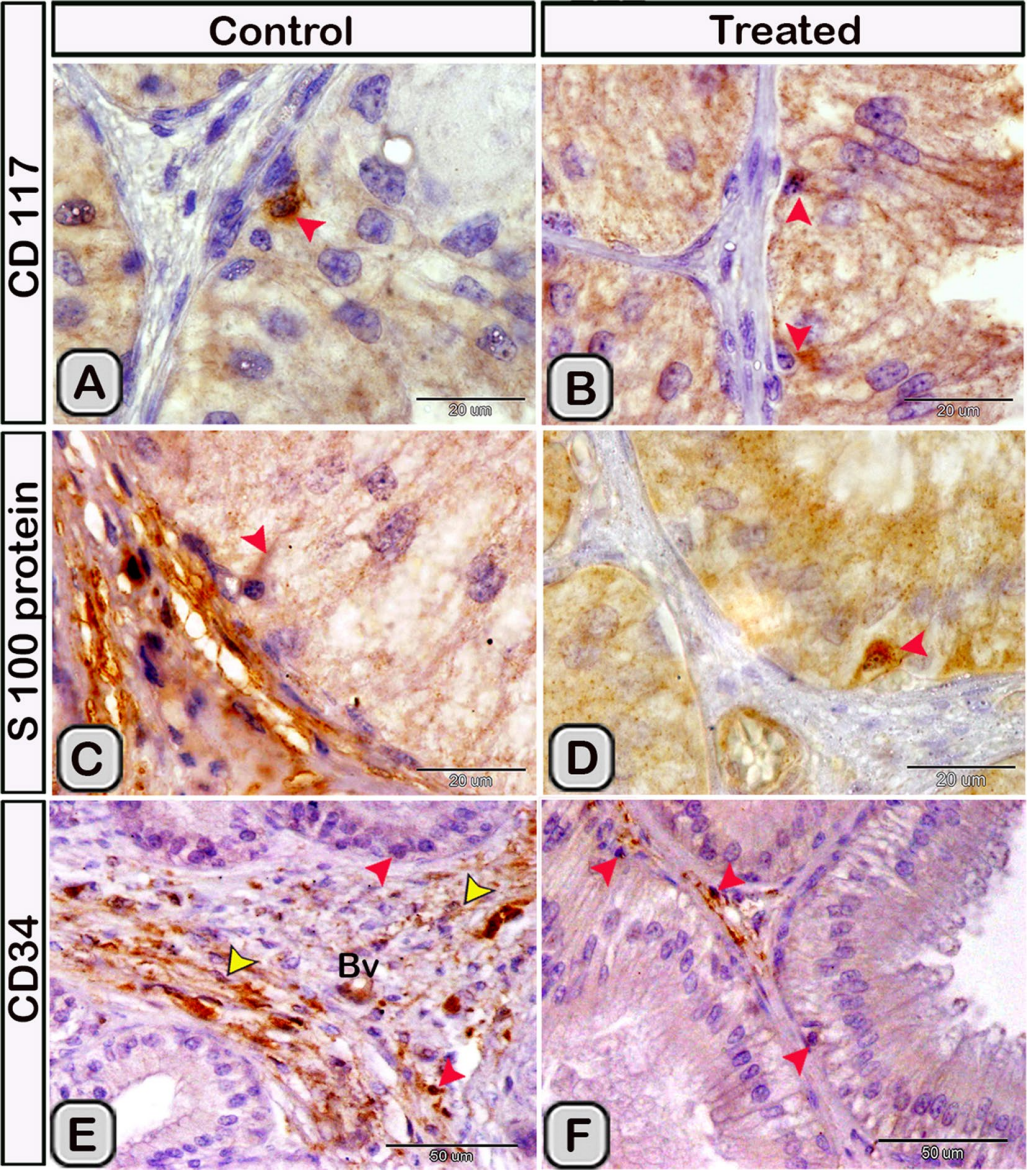
The effect of melatonin administration on the expression of CD117, S-100 protein and CD34 in dendritic cells (DCs). No obvious changes could be detected in the expression of CD117 **(A,B)** S-100 protein **(C,D)** and CD34 **(E,F)** (red arrowheads) in the melatonin treated group compared to the control. Notice: the expression of CD34 in telocytes (yellow arrowheads) endothelium of blood vessel (Bv).

**Figure 5:**
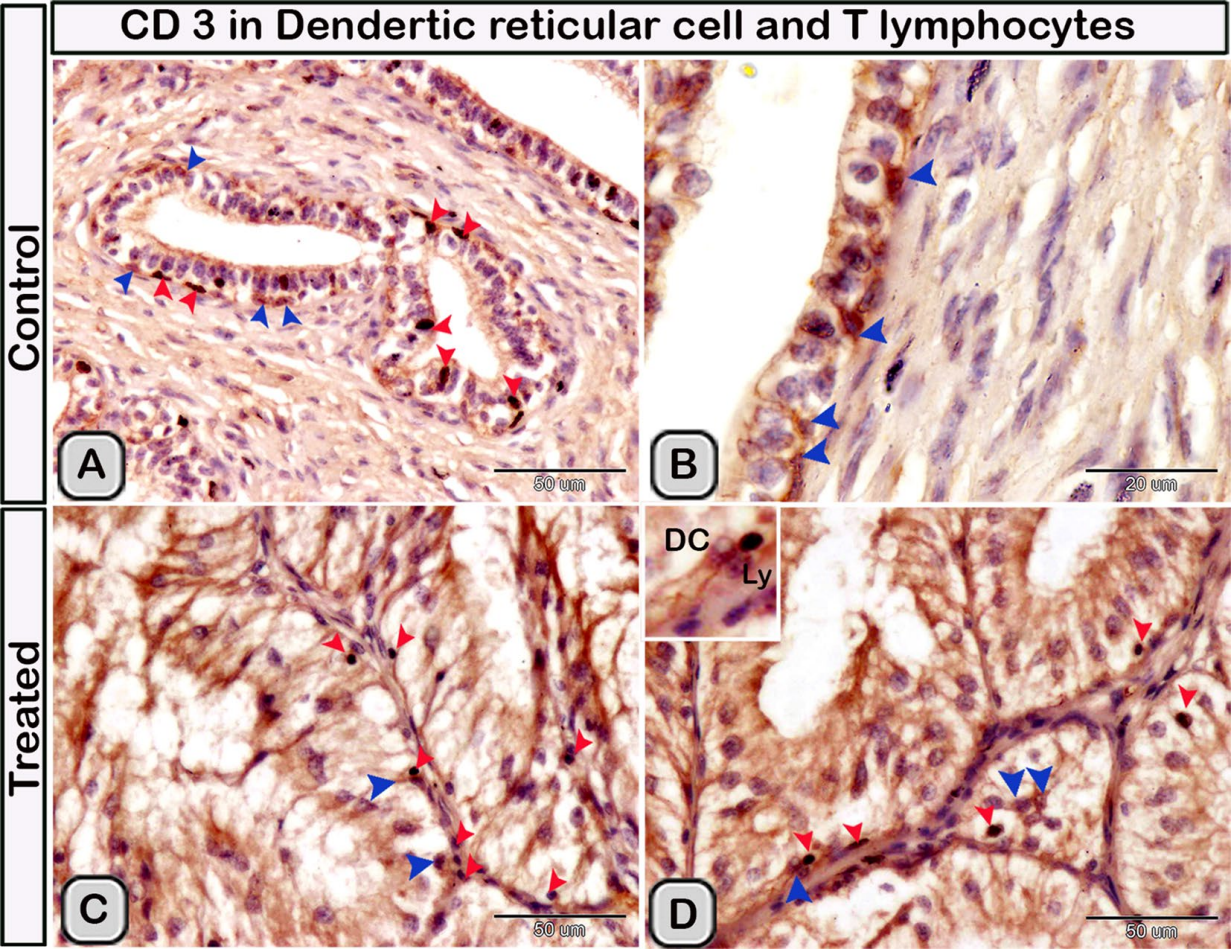
The effect of melatonin administration on the expression of CD3 in the dendritic cells (DC) and T-lymphocytes (Ly). **(A,B)** CD3 expressed in the DCs with its well defined processes (red arrowheads) and T-Lymphocytes (blue arrowheads) in the control groups. **(C,D)** The immunoreactivity for CD3 was more abundant in melatonin treated groups.

**Figure 6:**
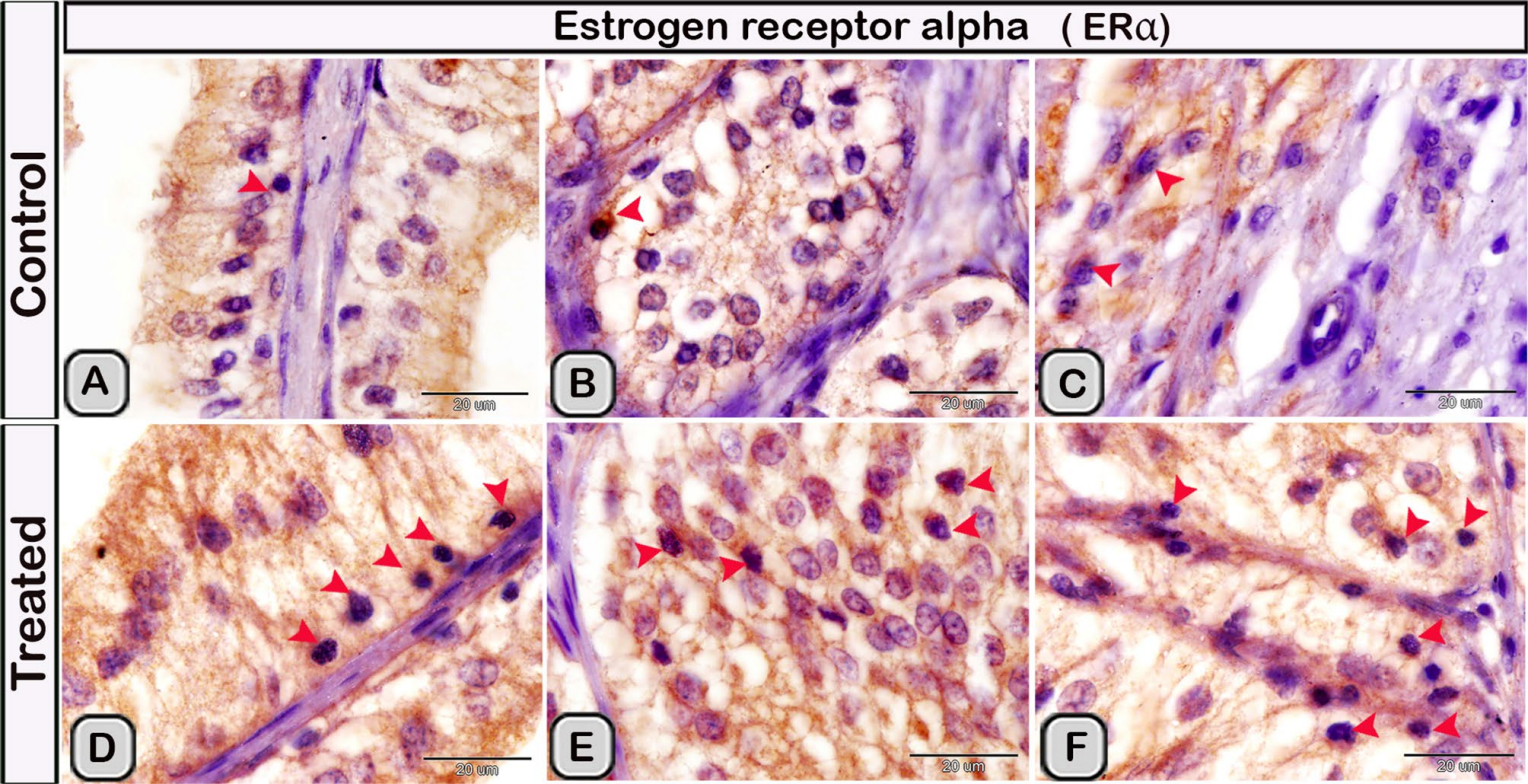
The effect of melatonin administration on the expression of estrogen alpha receptors in the dendritic cells (red arrowheads). **(A,B)** Expression of estrogen alpha receptor in the epithelium, **(C) **the expression in the interstitium of the control groups. **(D,F)** the expression of estrogen alpha receptor (ER-α) increased obviously in the melatonin treated groups.

**Figure 7:**
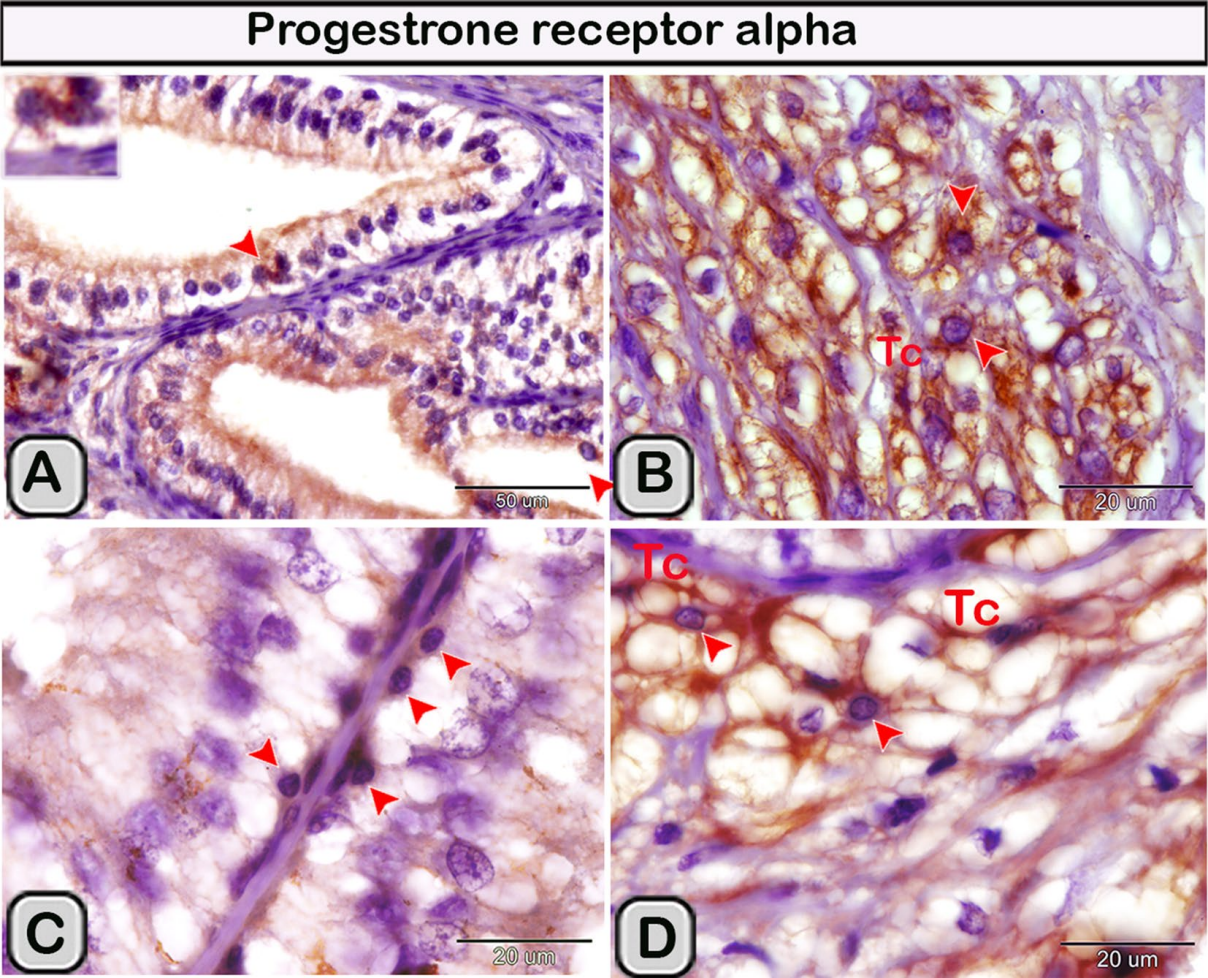
The effect of melatonin administration on the expression of progesterone in the DCs (red arrowheads). **(A,B)** Expression of progesterone receptor in the DCs in the epithelium and the interstitium of the control groups respectively. **(C,D)** The expression of progesterone receptors increased obviously in the melatonin treated groups. Notice: the close relation between the DCs (red arrowheads) and TCs (telocytes).

**Figure 8:**
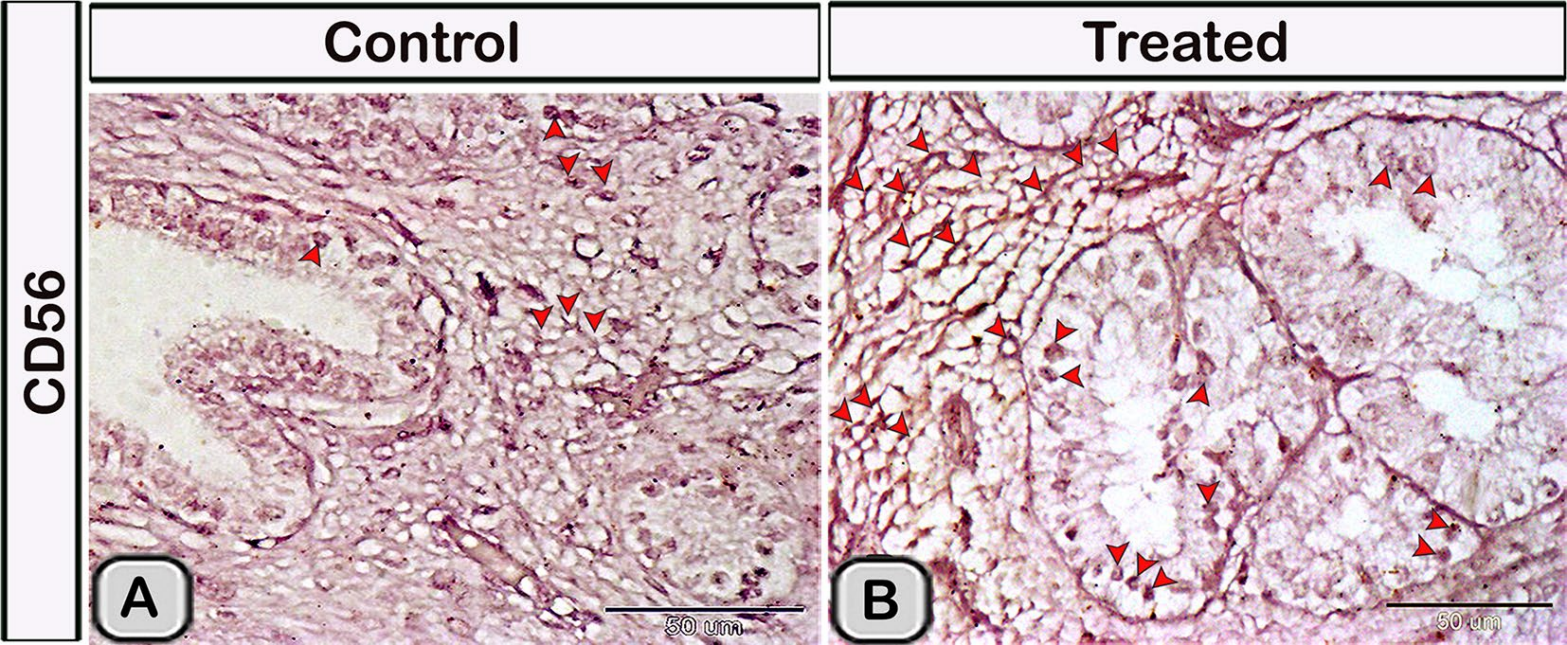
The effect of melatonin administration on the expression of CD56 in the DCs. **(A)** Expression of CD56 in the DCs lining the epithelium and the interstitium of the control groups. **(B)** the expression of CD56 was more abundant in the melatonin treated groups. The expression of CD56 in the interstitial DCs was branched and anastomsing similar to a continuous network.

**Figure 9:**
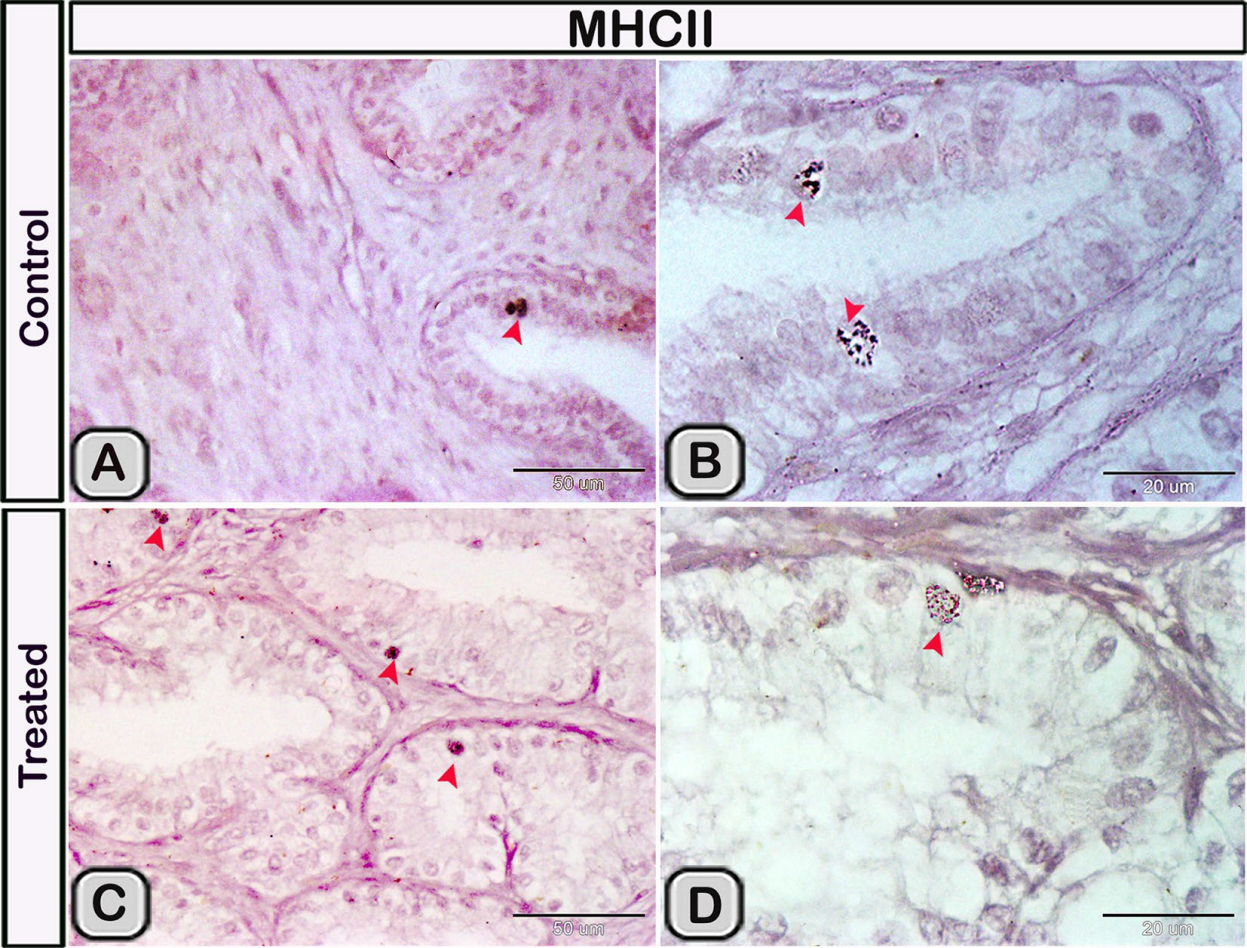
The effect of melatonin administration on the expression of MHC-II in the DCs. **(A,B)** The endosomal compartments of the dendritic cells showed positive immunoreactivity for MHC-II. **(C,D)** Expression of the MHCII in the endosomal compartments of the DCs was more abundant after melatonin administration (red arrowheads).

Immunohistochemical staining was performed to characterize the dendritic cells in the seminal gland of the control and melatonin treated groups. The dendritic cells showed positive immunoreactivity for CD117/c-kit (Fig. 4A,B), S-100 proteins (Fig. 4C,D) and CD34 (Fig. 4E,F). CD3 immunoreactivity were demonstrated in the T- lymphocytes which characterized by their small size and they were round to oval in shape. The immunoreactivity was also investigated in DCs with its well-defined processes (Fig. 5A,B). In the melatonin treated groups we demonstrated an obvious increase in the number of the positive immunoreactive cells to CD3 (Fig. 5C,D and Table 2). The immunoreactivity for estrogen receptor (ER-α) and progesterone demonstrated in the lining epithelium and the interstitium of the seminal gland (Figs. 6A–C, 7A,B). However, in the melatonin treated groups DCs showed marked increase in the expression of estrogen receptor (ER-α) and progesterone (Figs. 6D–F; 7C,D and Table 2). Furthermore, we examined the expression of two specific markers: CD56 and MHC-II (major histocompatability class II). DCs with its well-defined processes showed positive immunoreactivity for CD56 in the lining epithelium and the interstitium of the seminal gland (Fig. 8A). However, the expression of CD56 and MHC-II in the dendritic cells was abundant in the melatonin treated groups compared to the control (Figs. 8B, 9A,B). Expression of the MHCII in the endosomal compartments of the DCs was more numerous compared to the control ones (Fig. 9C,D).

#### Ultrastructure of the dendritic cell

**Figure 10:**
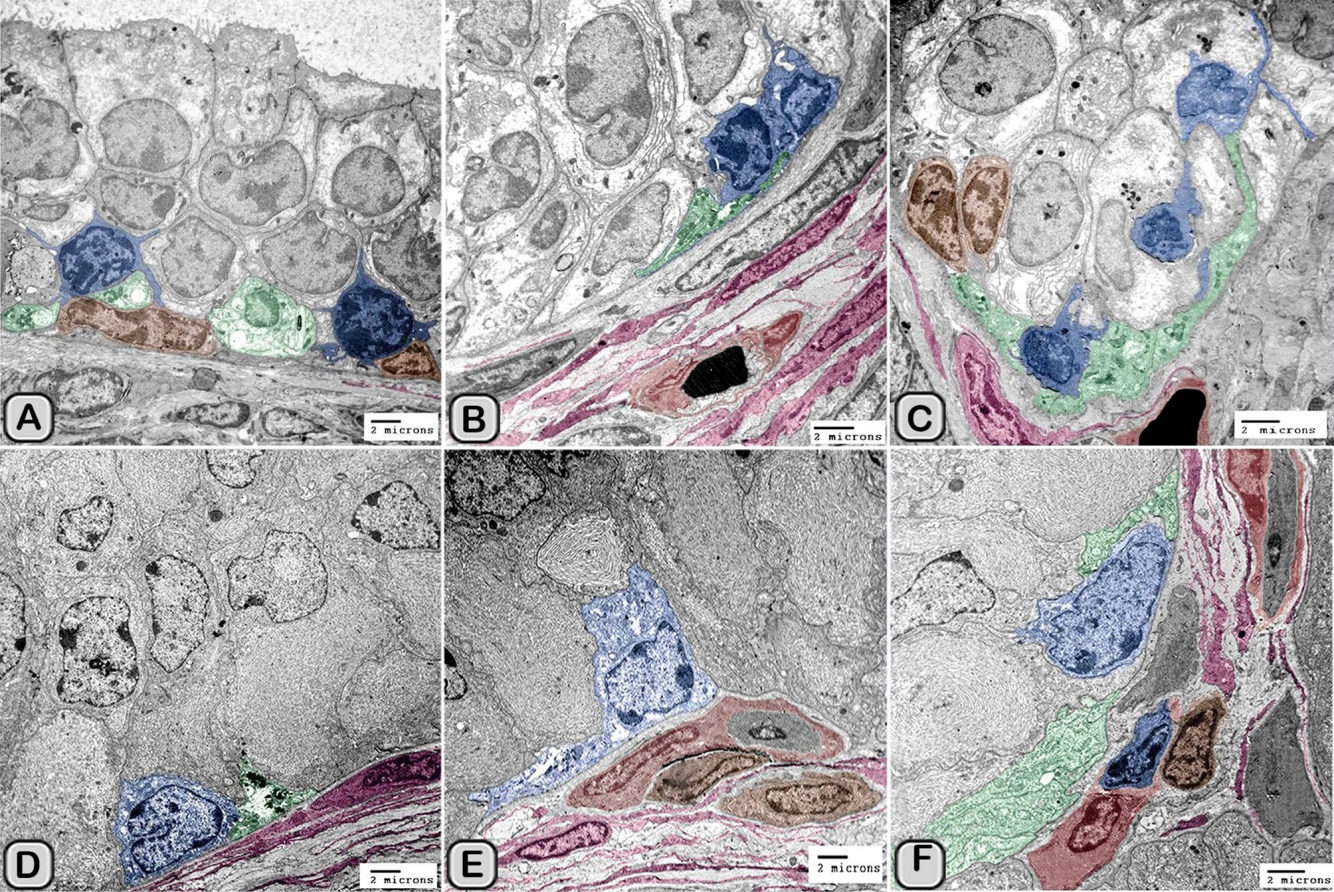
Digitally colored transmission electron microscope images of the dendritic cells in the seminal vesicle of the soay ram. **(A–C)** General morphology of the dendritic cells in the glandular epithelium lining the seminal vesicle of the control groups. The dendritic cells (blue) had a deeply indented nucleus and a great amount of the peripheral heterochromatin (HC). The DCs (blue) were observed in association to the T-lymphocytes (brown), nerve fibers (green) and telocytes in the interstitium (pink). **(D–F)** General morphology of the DCs in the glandular epithelium lining the seminal vesicle of the melatonin treated groups. The nucleus of the DCs (blue) was euchromatic (EC) **(D–F)**. DCs varied in shape which could be large polyhedral **(D,E)** or triangular **(F)**. DCs were closely associated with lymphoblast (brown) and telocytes (pink).

**Figure 11:**
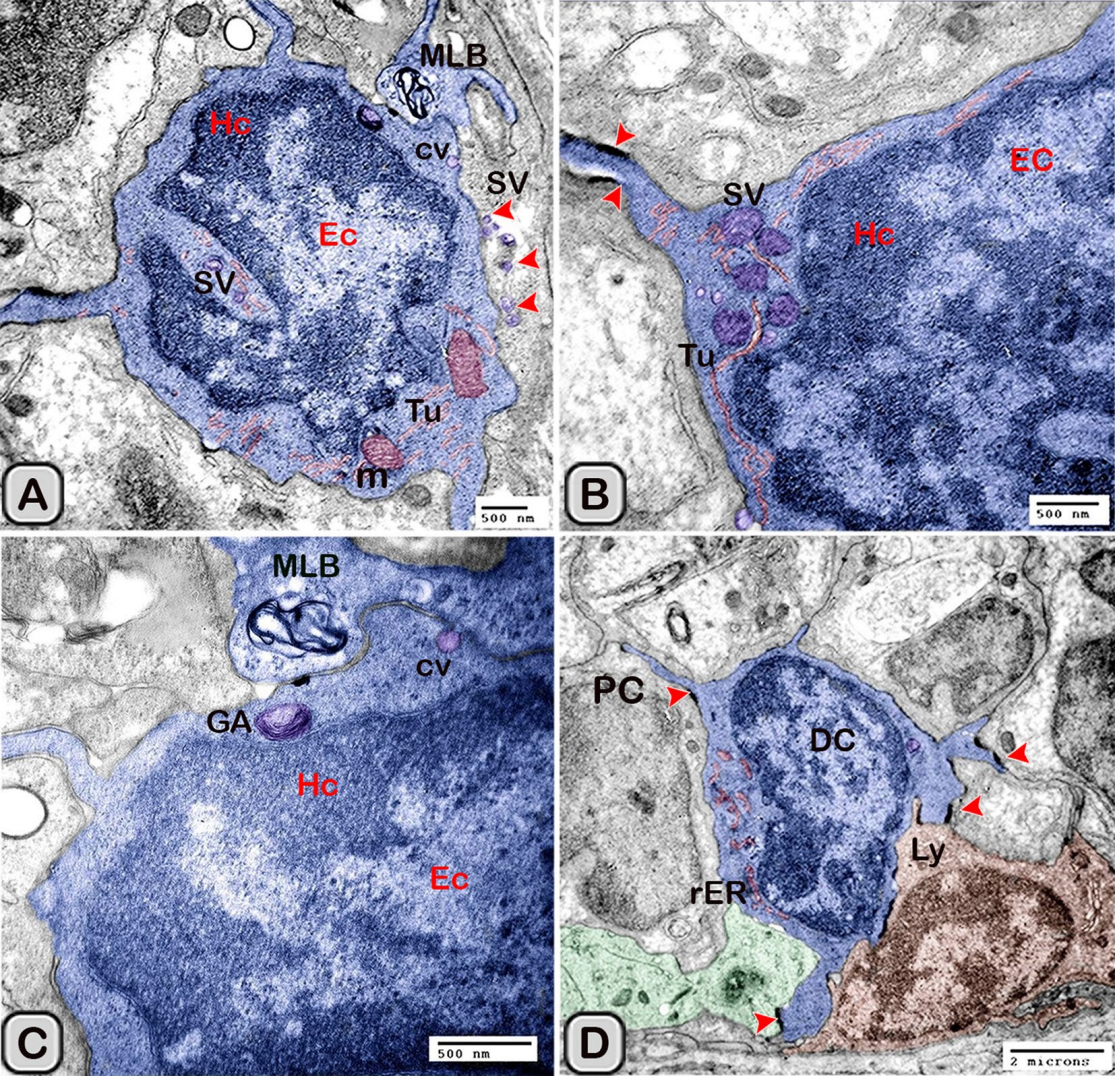
Digitally colored transmission electron microscope images of the DCs in the seminal vesicles of control groups. (**A–C**) The cytoplasm of the DCs (blue) contained relatively few cell organelles including the mitochondria (m), Golgi apparatus (GA), and short cisternae of the rER (arrowhead). The endosomal tubulo-vesicular system including the cavolae (cv), secretory vesicles (SV, red arrowhead) multilamellar bodies (MLB), numerous tubules (Tu) were observed. (**D**) The intercellular junctions such as the desmosomal junction (arrowheads) with the adjacent cells including T-lymphocytes (brown), principal cell (PC) lining the seminal vesicle and nerve fiber (green) were investigated. *HC* heterochromatin, *EC* euchromatin.

**Figure 12:**
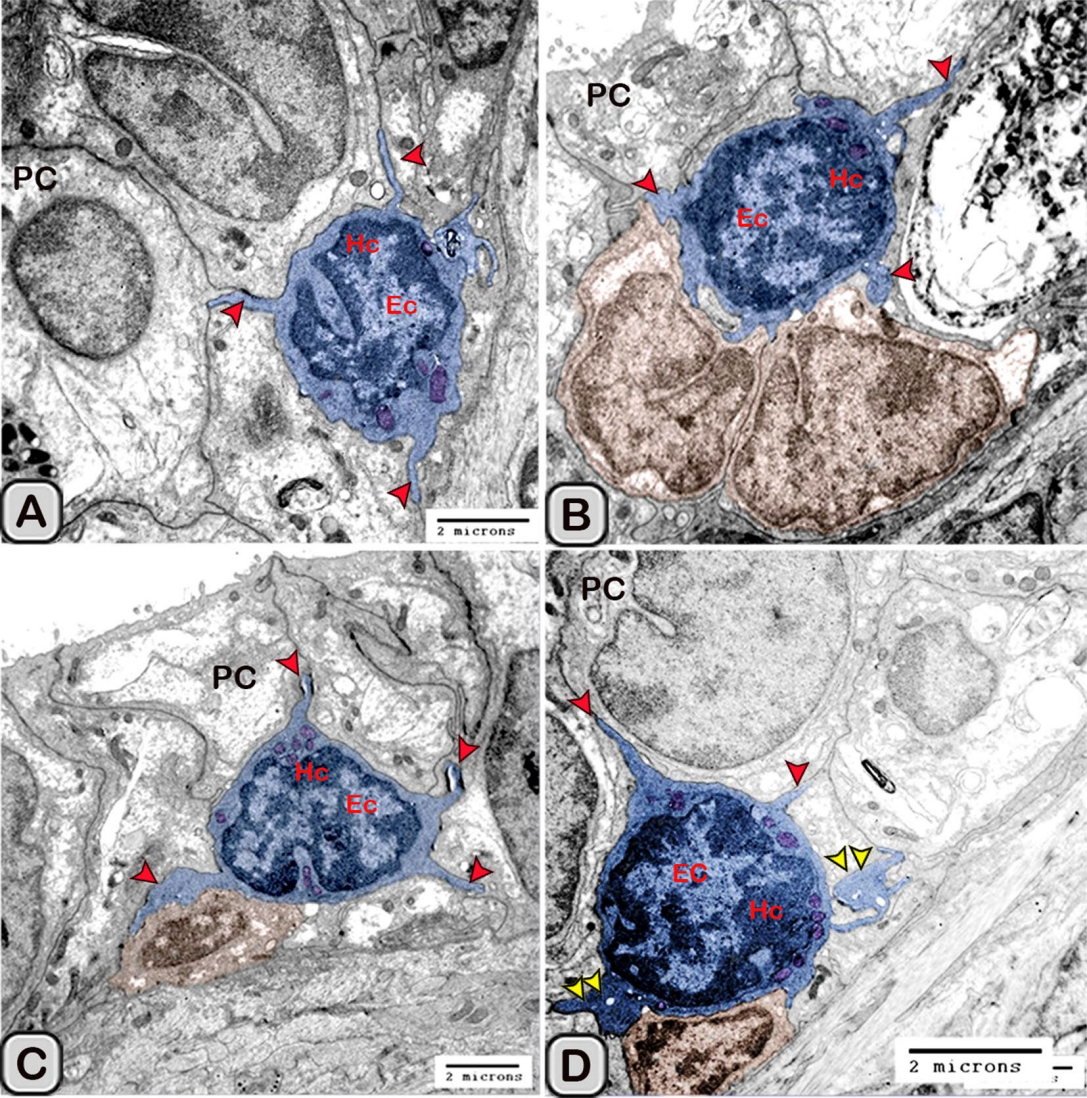
Digitally colored transmission electron microscope images of the DCs in the seminal vesicles of control groups. **(A–D)** DCs (blue) varied in shape which could be polyhedral, round or triangular. **(A–C)** The DCs located at the basement membrane or near from the basement membrane and in close association to T-lymphocytes (brown). **(A–C)** The cytoplasmic cell processes of dendritic cells were long (red arrowheads) and thick bulbous like cell processes (**D**, yellow arrow heads). *HC* heterochromatin, *EC* euchromatin, *PC* principal cell.

In addition to the immunohistochemical examinations, transmission electron microscopy (TEM) was performed to identify and characterize the dendritic cells in the seminal gland of Soay ram. TEM demonstrated the presence of dendritic cells (DCs) interdigitating between the epithelial cells lining the seminal gland through their long dendrites. The nucleus of the DCs in the untreated groups retained deeply indented nucleus with a peripheral distribution of heterochromatin. We observed a close association between the DCs, T-lymphocytes and nerve fibers (Fig. 10A–C). Dendritic cells located at the basement membrane (Fig. 10A,B) or free mobile among the glandular epithelium lining the seminal gland (Fig. 10C). In the melatonin treated groups the activated dendritic cells contain a large cell body and euchromatic nucleus and cells appeared variable in shapes including rounded, polyhedral and triangular. The DCs lining the seminal gland were observed closely related to telocytes (TCs) with its telopodes (Tp), nerve fibers, lymphoblast and the blood vessels in the interstitium of the gland (Fig. 10D–F). We observed that the cytoplasm of the dendritic cells contained relatively few membranous cell organelles including the mitochondria, Golgi apparatus and short cisternae of rough endoplasmic reticulum (rER) which arranged close to the nucleus (Fig. 11A–D). The endosomal tubulo-vesicular system observed in the cytoplasm of the dendritic cells, which including the coated vesicles, multilamellar bodies (MLB), numerous tubules and vesicles (Fig. 11A–C). Among the morphological features of the dendritic cells were the desmosomal intercellular junctions with the adjacent principal cells lining the seminal gland and the associated T-lymphocyte (Fig. 12A–C). The dendritic cells demonstrated in a close contact to one or two T-lymphocytes (Fig. 12B–D). The cytoplasmic cell processes of dendritic cells were long and thick bulbous like cell processes (Fig. 12C,D).

**Figure 13:**
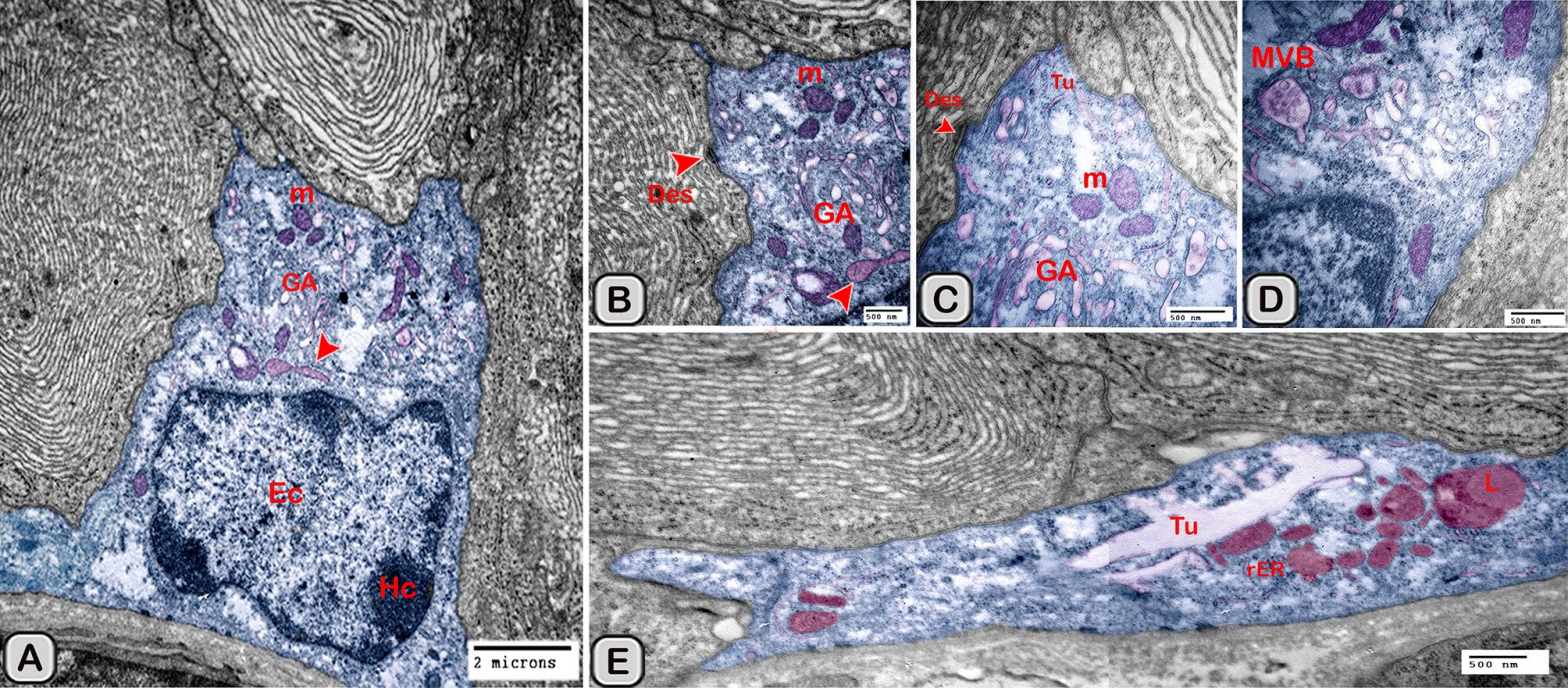
Digitally colored transmission electron microscope images of the activated DCs in the melatonin treated group. **(A)** General view of an active polyhedral dendritic cell with an abundant cell organelles and euchromatic nucleus (Ec). **(B–D)** Higher magnification showing abundant mitochondria (m) and well developed Golgi apparatus (GA). **(B–E)** Birbeck granules (red arrowheads), multivesicular bodies (MVB), tubules (Tu) and phagolysosomes (L) were observed. Desmosomal cell junction with the neighboring cell (red arrowhead, Des) was observed.

**Figure 14:**
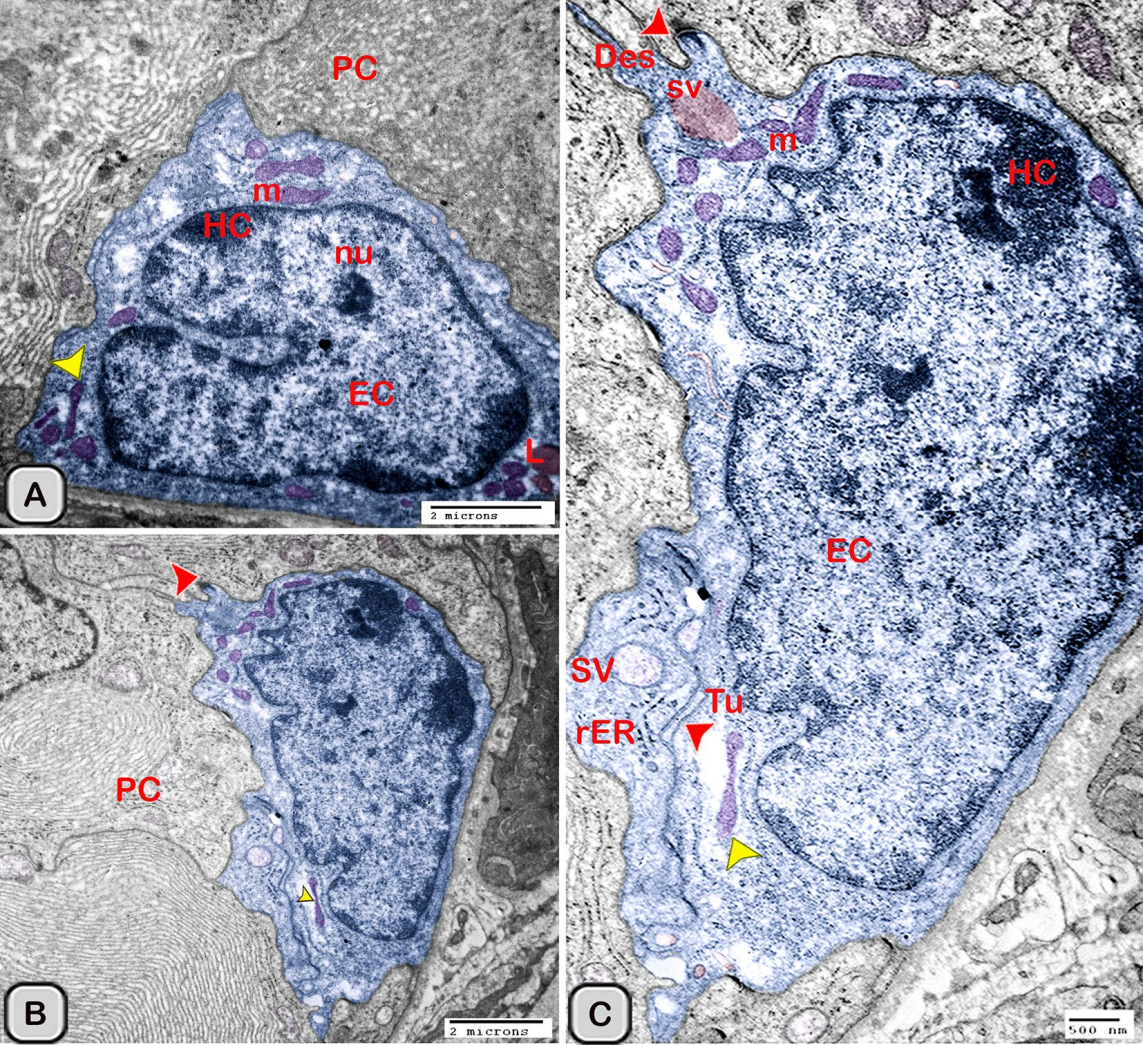
Digitally colored transmission electron microscope images of the activated DCs in the melatonin treated group. **(A,B)** An active polyhedral and triangular shaped dendritic cell with abundant cell organelles and euchromatic nucleus (Ec) with a distinct nucleolus (nu) were investigated. **(C)** Higher magnification showing abundant mitochondria (m), rER, secretory vesicles (Sv), tubules (Tu), lysosomes (L) and Birbeck granules (yellow arrowheads). Moreover, the cell processes were thin and short and desmosomal junctions (Des, red arrowheads) were observed.

The size (cross sectional area) of the dendritic cells increased significantly when compared to the control groups (Table 2). The nucleus of the DCs was euchromatic and contained a prominent nucleolus (active cell), in addition it was larger and more indented compared to the control group (Figs. 13 and 14). We demonstrated an increase in the secretory activity of the dendritic cells after the melatonin treatment. There was an obvious increase in the number of mitochondria, cisternae of rER and a well-developed supranuclear Golgi apparatus with its secretory vesicle (Fig. 13A–D). The endosomal- lysosomal system was more developed than the control groups and composed of numerous tubules and vesicles, multivesicular bodies and phago- lysosomes (Fig. 13D,E). In addition, a rod-shaped Birbeck granule (club-shaped) demonstrated in the cytoplasm nearby the nucleus of the dendritic cells (Fig. 14A–C). The cell process was thinner and shorter than those observed in the control groups. The desmosomal cell junctions were more obvious between the dendritic cells and the neighboring principal cells lining the seminal vesicle (Fig. 14B,C).

**Figure 15:**
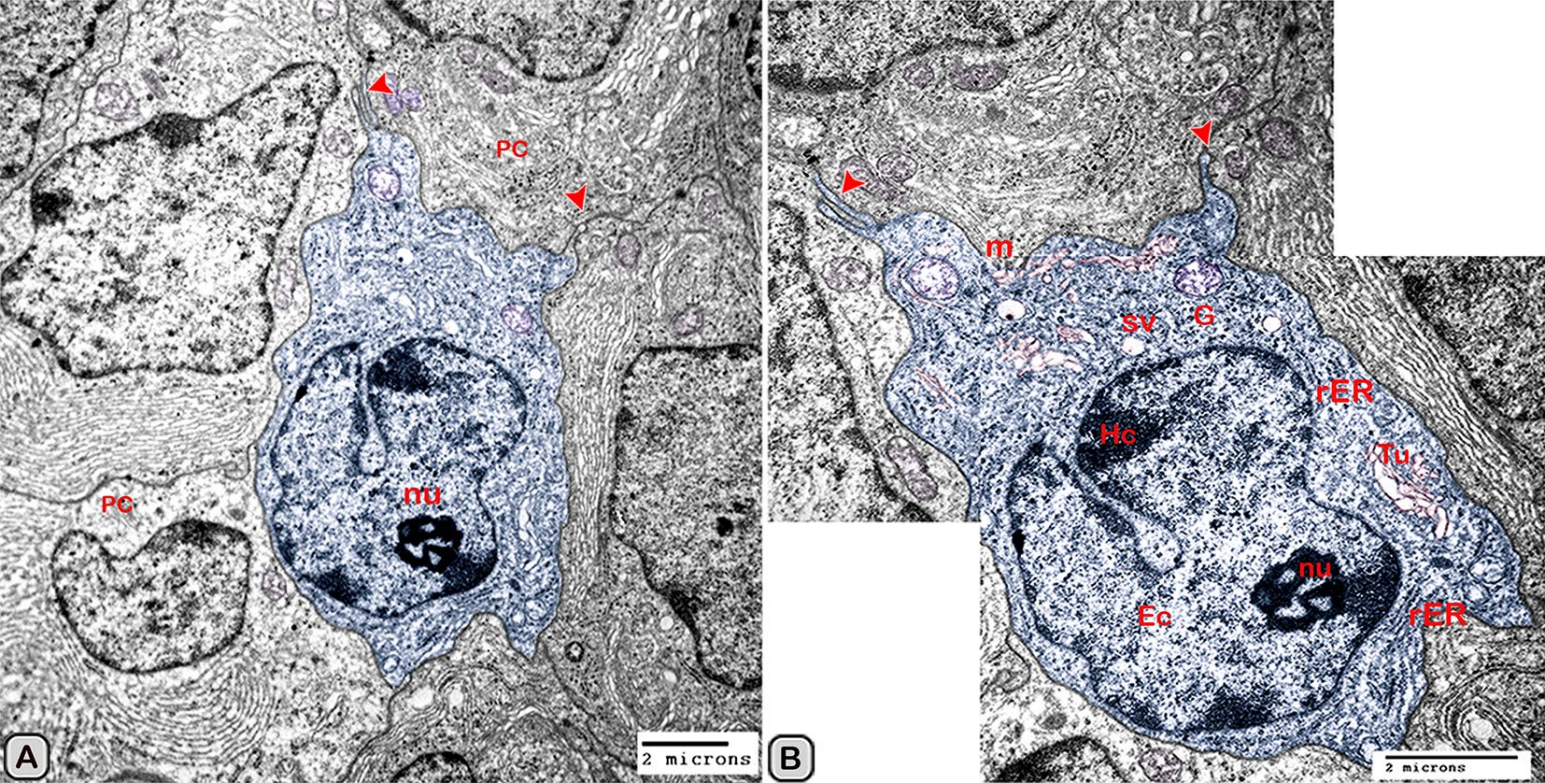
Digitally colored transmission electron microscope images of the free movable immature dendritic cells. **(A,B)** The nucleus was euchroamtic (Ec) and deeply indented with a distinct nucleolus (nu). **(B)** The cytoplasm consisted of well-developed rER, a great amount of the dense granules (G), secretory vesicles (SV) a moderate number of mitochondria (m) and ill-developed tubule-vesicular system (Tu). The cell processes were short and thin or thick (red arrowheads). *HC* heterochromatin, *EC* euchromatin, *PC* principal cell.

The free movable dendritic cells showed different stages of maturity in the melatonin treated groups. The nucleus of immature free movable dendritic cells was euchroamtic and deeply indented with a distinct nucleolus. The cytoplasm consisted of well-developed rER, a great amount of the dense granules, secretory vesicles, a moderate number of mitochondria and an ill-developed tubule-vesicular system. The cell processes were short and thin or thick (Fig. 15A,B).

**Figure 16:**
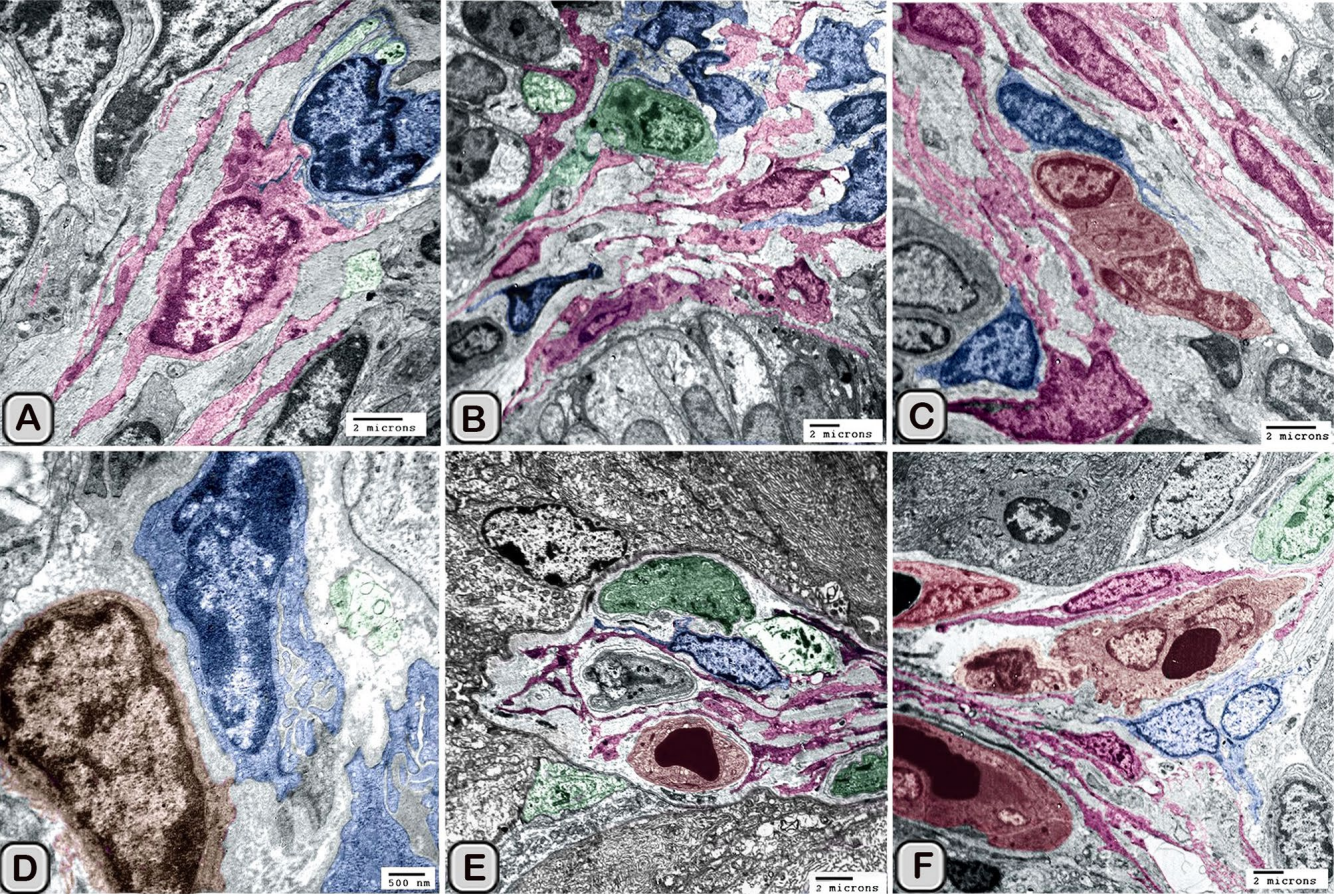
Digitally colored transmission electron microscope images of the Interstitial DCs in the control **(A–C)** and melatonin treated groups **(D–F)**. DCs process (blue) form hetercellular junctions with telocytes and its telopodes (pink). In addition, DCs were observed in a close contact to T-Lymphocytes (brown), Schwann cell, nerve fibers (green) and blood vessels (reddish brown).

Interstitial dendritic cells were demonstrated in the interstitium of the control and melatonin treated groups. Interstitial DCs were observed in close contact with telocytes and its telopodes (Tps), T-Lymphocytes, Schwann cell and blood vessels (Fig. 16A–F). The cross sectional area of the interstitial DCs varied between the control and melatonin treated groups, however they are not significantly different between the two groups (Table 2)

**Figure 17:**
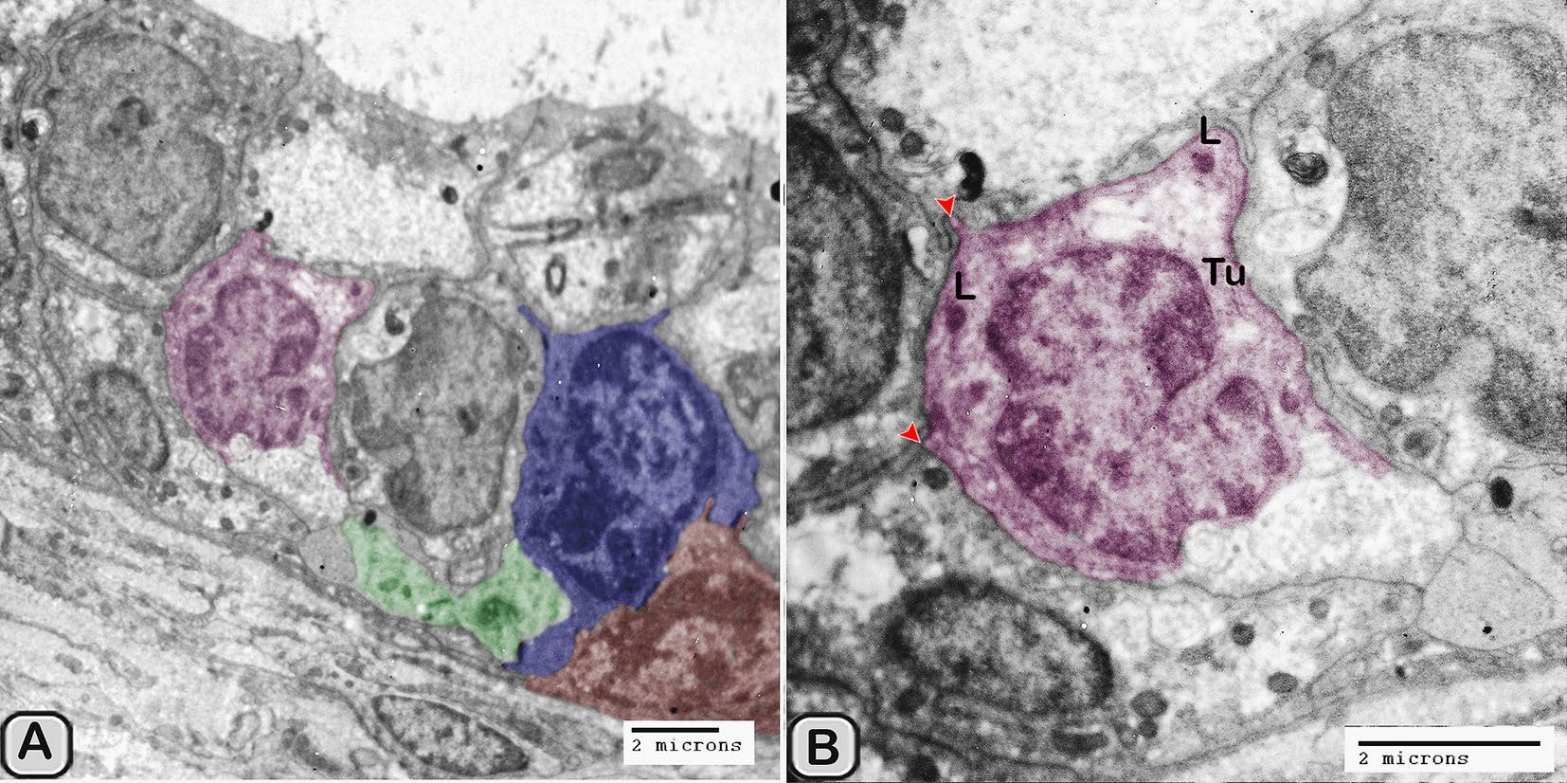
Digitally colored transmission electron microscope images of Macrophage (violet) among the epithelium of Soay ram seminal gland in the control groups. **(A,B)** macrophages were observed nearby the dendritic cells (blue), T-lymphocytes (brown) and nerve fibers (green). The nucleus was kidney shape and their cytoplasm contains lysosomes (L) and ill-developed endosomal tubular system (Tu).

**Figure 18:**
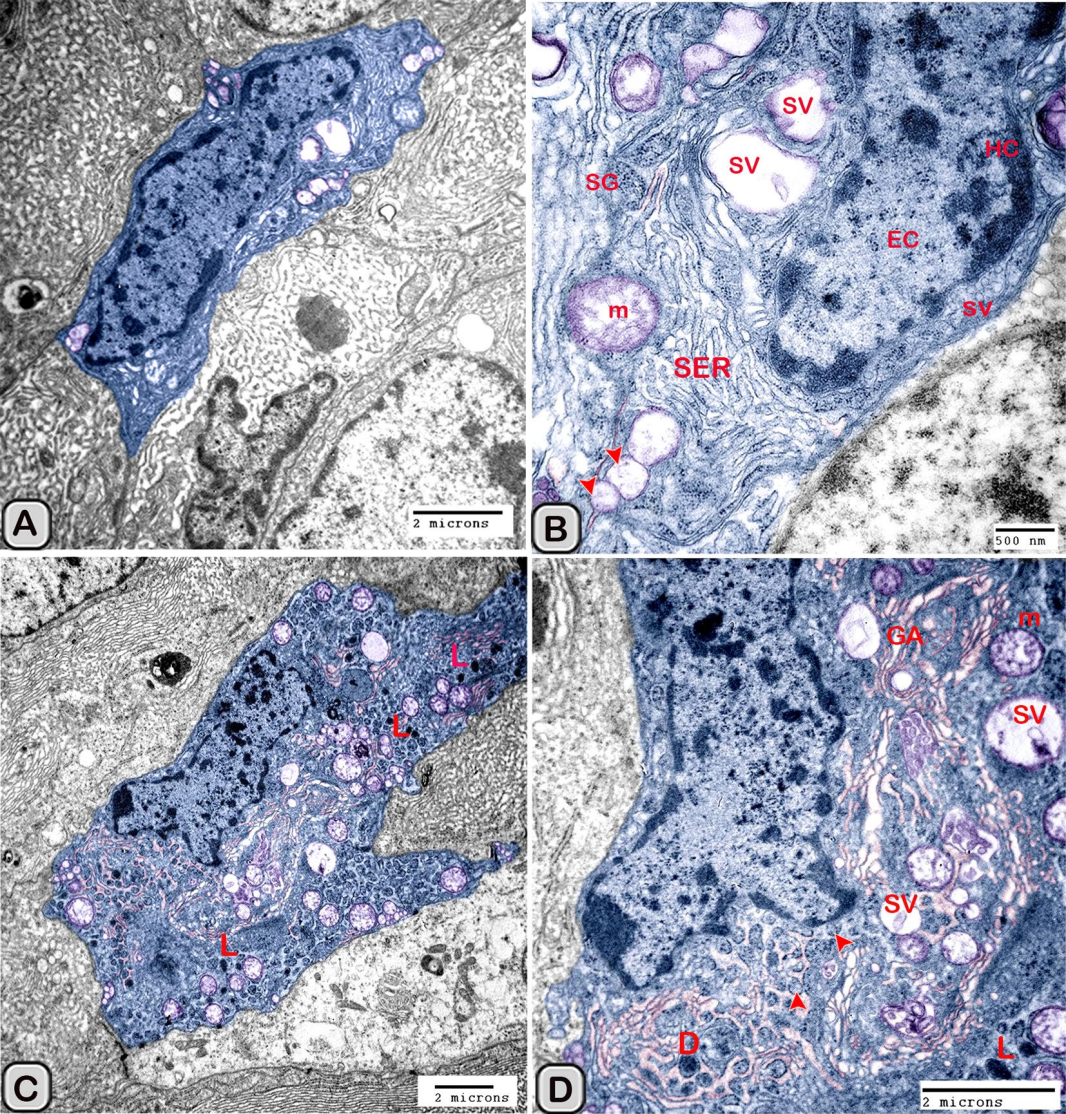
Digitally colored transmission electron microscope images of Macrophage among the epithelium of Soay ram seminal gland in the melatonin treated groups **(A–D)**. **(A,B)**The cytoplasm contained a well-developed tubulo-vesicular endosomal system (Tu), smooth endoplasmic reticulum (SER), secretory vesicles (Sv) and a well-developed Golgi (GA). In addition, the dense bodies (DB) and lysosomes (L) were more obvious compared to the control ones.

Macrophages were also demonstrated among the epithelium lining the seminal gland in the control groups and nearby the dendritic cells (Fig. 17A,B). The macrophages nucleus was kidney shaped and the cytoplasm contained lysosomes and ill-developed tubular endosomal system. In the melatonin treated groups, the macrophages appeared activated (Fig. 18A–D). The cytoplasm contained a well-developed tubulo-vesicular endosomal system, smooth endoplasmic reticulum (SER), secretory vesicles, mitochondria and a well-developed Golgi apparatus. In addition, the dense bodies and lysosomes were more evident compared to the control ones.

## Discussion

In the current study, we identified and characterized the dendritic cells in the glandular epithelium and the surrounding interstitial tissue of the Soay ram seminal glands. DCs have a unique morphological structure and functional properties when they compared to other antigen presenting cells such as macrophages as they are strong naïve T-lymphocytes stimulators via peptide-presenting MHC (major histocomptability) class II molecules^57–59^. In the present work, we identified the dendritic cells morphologically by conventional stains, immunohistochemistry and ultra-structure. They are characterized by their typical cytoplasmic processes (dendrites), indented nuclei and by their close association to T- lymphocyte cells. Dendritic cells (DCs) and macrophages are highly dynamic cells which show changes in their function, distribution and phenotype. Dendritic cells are critically involved in the initiation and modulation of an appropriate immune response by linking innate to adaptive immune responses ^12^. Interstitial dendritic cells were demonstrated in the interstitium of the seminal gland in close association to the blood vessels, telocytes and nerve fibers. Telocytes had been identified in the interstitium of many organs; to react with the interstitial cells and immune cells^60,61^. We demonstrated a close association between the dendritic cells and the nerve fibers. These areas of contact contain important neuromediators including substance P and calcitonin-gene related peptide (CGRP) which are critical for the crosstalk between the nervous and immune systems^62^. The current study demonstrated a stimulatory effect exerted by the melatonin on the dendritic cells, such as increasing the number and the secretory activity of DCs observed by TEM. The endosomal- lysosomal system was more developed in the melatonin treated groups compared to the control ones. The current result revealed that the endosoaml system consisted of numerous vesicular and tubular compartments, cavolae, multivesicular bodies and lysosomes. This pathway is highly dynamic and involved in segregating the components fated for degradation in lysosomes or recycling to the plasma membrane or Golgi apparatus^63,64^. The endosomal system had a role in the endocytosis of the antigen for processing, then transport the peptide/MHC class II to the plasma membrane. Therefore, the endosomal tubulo-vesicular system acts as sites for the activation of T-lymphocyte cell. Moreover, it acts as a storage site for several immunoregulatory factors which secreted form the DCs^65,66^. We demonstrated Birbeck granules, which are club shaped structure in the melatonin treated groups. Birbeck granules composed of Langerin protein (CD207), which found in the endosomal recycling compartment^67^. Birbeck granules play an important role in antigen processing as they allow internalization of antigens until their presentation to T-Lymphocytes^68^.

Melatonin exerts its action through M1 and M2 membrane receptors^69^. Melatonin receptors had been identified in the ram testis, epididymes, vas deferens and seminal vesicles^70^. Melatonin is an immunomodulator, which produced by the immunocompetent cells and lymphocytes^71^. Moreover, melatonin enhances the proliferation and maturation of all immune cells including T and B lymphocytes, granulocytes and monocytes^72^. In addition, melatonin increases testosterone level during the non-breeding season in the blood plasma, the sperm quality, the testicular parameters, the spermatogenesis and fertility of rams. In our previous studies we investigated the enhancement role of melatonin on the secretory activity of the seminal gland and its glandular epithelium. Therefore, we concluded that the effect of melatonin on DCs could be directly through the melatonin receptor or indirectly through the increase in testosterone level.

In the current work, we demonstrated that the dendritic cells showed positive immunoreactivity for CD117 and CD34. CD117/c-KIT is a tyrosine-kinase receptor expressed on the outer surfaces of hematopoietic cells and stem cells (Martin et al., 1990). Moreover, CD117 and CD 34 are used as a specific marker for telocytes in the seminal vesicles of Soay ram^39^. CD117 expression in most mature hematopoietic cells is absent during the final stages of differentiation. However, some subsets of dendritic cells express CD117 throughout their development^73^. CD34 is a marker for blood progenitor cells and many undifferentiated cells^74^. It is well known that CD3 is a marker of T- lymphocytes in all stages of maturation^75^. We demonstrated that CD3 expressed in the T-lymphocytes and the DCs. A unique feature of DCs was their ability to proliferate and activate MHC class II dependent which primarily involves CD3 and CD4 T cells. Afterwards, the proliferating T-cells were able to produce the effectors cytokines interleukin-4 and interferon^76^. S-100 is a calcium-binding protein which is widely expressed in a variety of cell, such as glial and Schwann cells of the nervous system, epidermal langerhans cells, melanocytes and telocytes in different organs^9,77,78^ mentioned that most of the immunoreactive cells to S-100 protein in the human uterine tube were related to the dendritic cells. In the current work, we observed an obvious increase in the number of DCs, which immunoreacted positively with estrogen receptor alpha (ER-α) and progesterone receptors. It is well known that the function of dendritic cells is highly controlled by sex steroid hormones. In addition, DCs express receptors for the steroid hormones which, acts as a primary target for their actions during infection^79^. Estrogen receptors, particularly ER-α have been identified in many different immune cell types including hematopoietic precursors, CD34+ human hematopoietic progenitor cells and DCs subsets^80,81^. Estrogens act directly through their receptors, on progenitor cells to regulate various facets of convential and plasmacytoid DCs^82–84^. CD56 (the neural cell adhesion molecule, NCAM) is expressed by many immune cell subsets including natural killer cells, monocytes, gamma delta T cells and dendritic cells It has been investigated that both plasmacytoid and myeloid DCs can adapt a CD56^**+**^ phenotype and acquire cytotoxic functions^85^. The current study demonstrated that CD56 expression was more abundant in the melatonin treated group in the epithelium and the interstitium. The expression of CD56 in the interstitial DCs was branched and anastomosing similar to a continuous network. CD56 had a vital role in the formation of preferential synapses between the CD56^**+**^ immune cells^85^. The MHCII molecule is expressed by all subtypes of DCs as well as macrophages and certain populations of B-cells^86^. The current study demonstrated that endosomal compartments were more numerous in the melatonin treated groups, indicating the activation of the DCs. We confirmed this observation utilizing the transmission electron microscopy.

In conclusion, we observed melatonin administration elicits a stimulatory action on the dendritic cells and macrophages of the Soay ram seminal gland. The current work supports that melatonin enhances the immune response through increasing the size, the number and the endosomal compartments of DCs and macrophages which may correlate to increased immunity. Dendritic cells have an emerging role in novel cancer therapies^87^. Therefore, Future research in this field should be done to ensure the effectiveness of melatonin on therapeutic causes under clinical conditions.

## Supplementary Information

Supplementary Figures.

## Data Availability

All data generated or analyzed during this study are included in this published article and its Supplementary Information files. The datasets used and/or analyzed during the current study are available from the corresponding author on reasonable request.

## Abbreviations

DCs: Dendritic cells
TCs: Telocytes
Tps: Telopodes
TEM: Transmission electron microscopy
rER: Rough endoplasmic reticulum
SER: Smooth endoplasmic reticulum
BVs: Blood vessels
