# Supplementary material for: Melatonin administration provokes the activity of dendritic reticular cells in the seminal vesicle of Soay ram during the non-breeding season
Source: Sci Rep. 2021 Jan 13;11:872. doi: 10.1038/s41598-020-79529-y (PMC7806754; doi:10.1038/s41598-020-79529-y)
Supplement: Supplementary file 1 — Supplementary Figures. [file 41598_2020_79529_MOESM1_ESM.pdf]

Melatonin administration provokes the activity of dendritic reticular cells in the seminal vesicle of Soay ram during the non-breeding season

Running title: Effect of melatonin on the dendritic cells

Hanan H. Abd-Elhafeez, A.H.S Hassan, Manal T. Hussein*

Department of Anatomy, Embryology and Histology, Faculty of Vet. Medicine, Assiut University, 71526, Egypt

Hanan H. Abd-Elhafeez and Manal T. Hussein are contributed equally to this work

Author Affiliations:

Hanan Hassan Abdel-Hafeez

Associate professor in Department of anatomy, embryology and histology, Faculty of Veterinary Medicine, Assiut University, Assiut (71516), Egypt. Email: hhnnzz91@aun.edu.eg. ORCID ID: https://orcid.org/0000-0002--2547-0709

Ahmed Hassan Sayed Hassan

Professor in Department of anatomy and histology, Faculty of Veterinary Medicine, Assiut University, Assiut (71516), Egypt

Department of anatomy, embryology and histology, Faculty of Veterinary Medicine, Assiut University, 71526, Assiut, Egypt

Email: ahassansayed@yahoo.com

Manal T. Hussein* (Corresponding author):

Lecturer of anatomy, embryology and histology, Faculty of Veterinary Medicine, Assiut University, Assiut (71516), Egypt

Tel: 00201010480875

ORCID ID: https://orcid.org/0000-0003-2801-6066

Email: manaltawfik22@gmail.com, manal.hussein@vet.au.edu.eg

CMEIAS color segmentation: (for all supplementary images)

Negative images performed by using CMEIAS color segmentation **(CMEIAS color segmentation** is a free, improved computing technology), http://www.mybiosoftware.com?cmeias-color-segmentation-1-0-segment-analyze-foreground-objects-complex-images.html

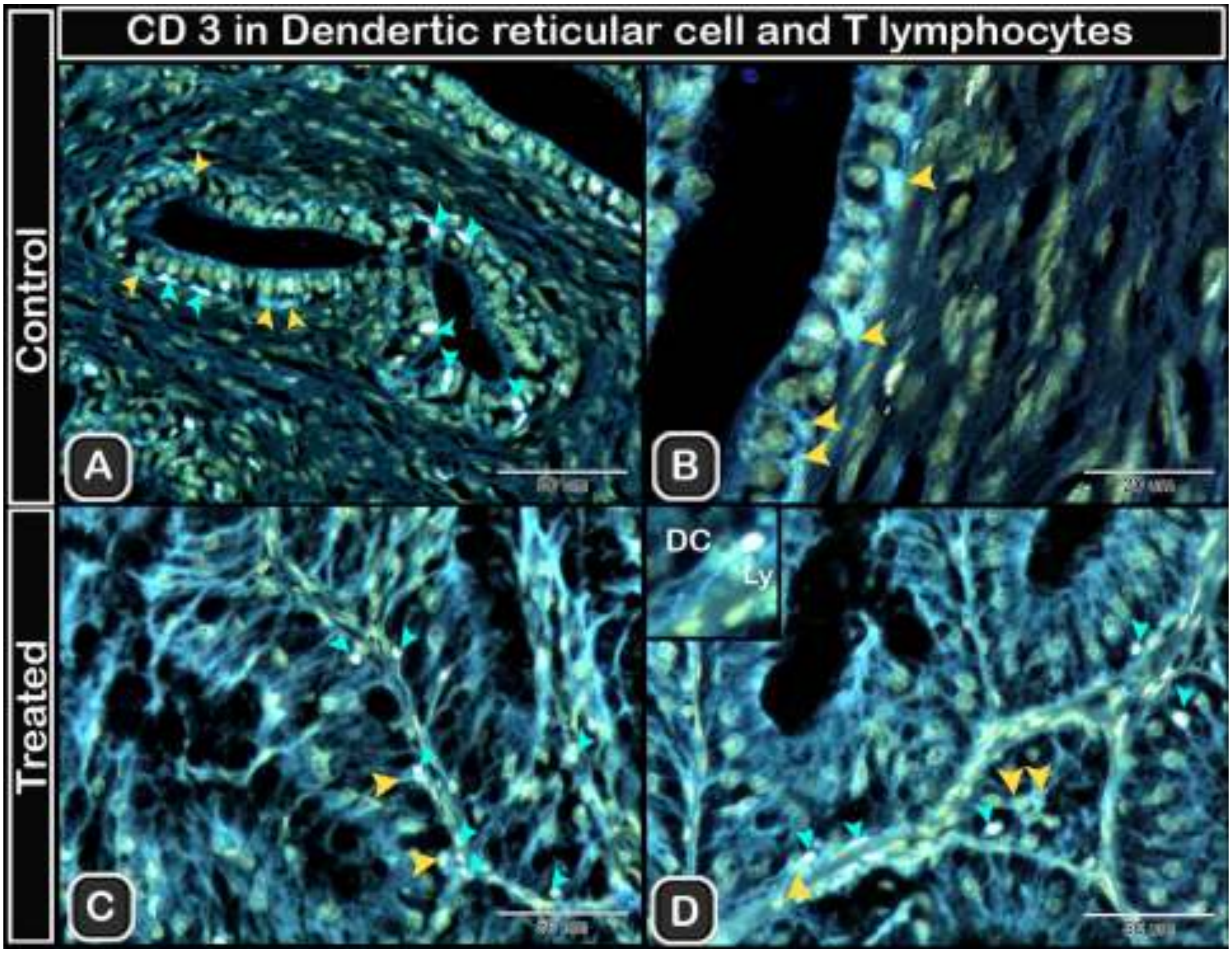

Supplementary legend 1: Negative images for organization of CD3 positive DCs (orange arrowheads) and T-Lymphocytes (green arrowheads) in Soay ram seminal gland. (A, B) control groups (C-D) melatonin treated groups by using CMEIAS Color Segmentation.

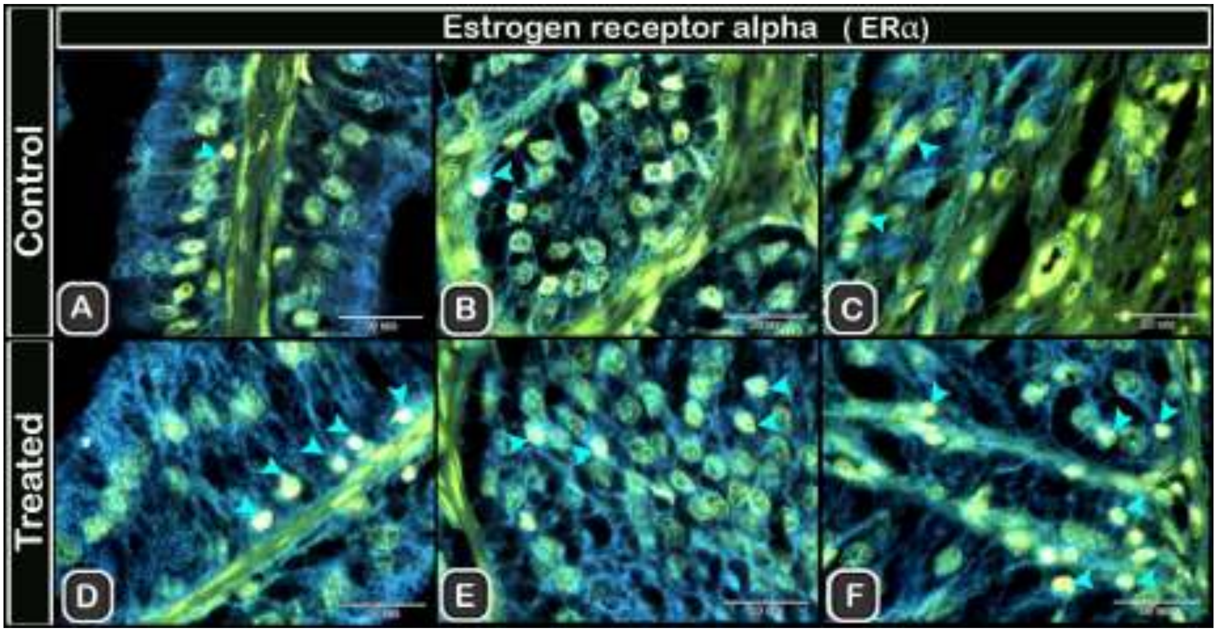

Supplementary legend 2: Negative images for organization of estrogen alpha-receptor positive DCs (green arrowheads) in Soay ram seminal gland. (A-C) control groups (D-E) melatonin treated groups by using CMEIAS Color Segmentation.

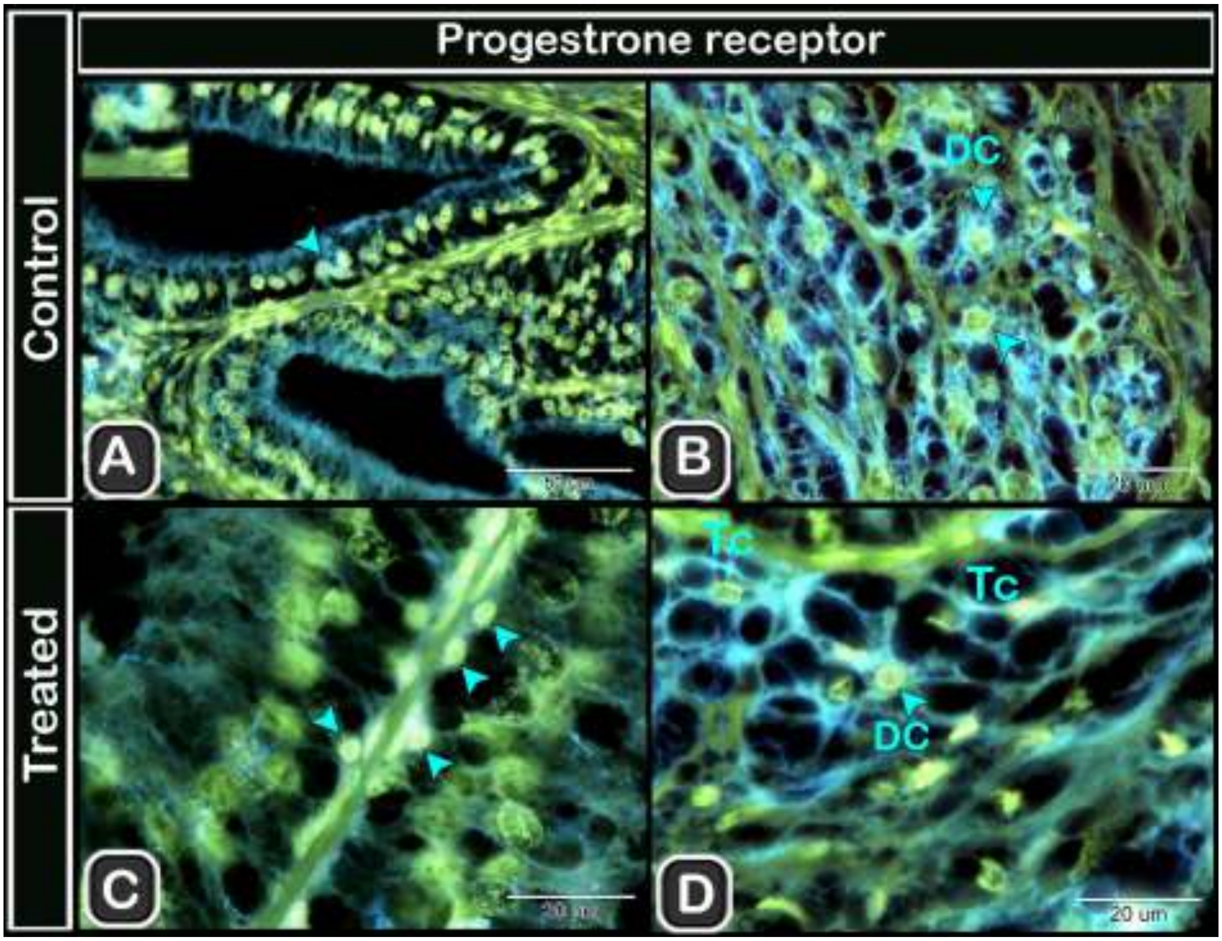

Supplementary legend 3: Negative images for organization of progesterone receptor positive DCs (green arrowheads) in Soay ram seminal gland. (A-B) control groups (C-D) melatonin treated groups by using CMEIAS Color Segmentation.

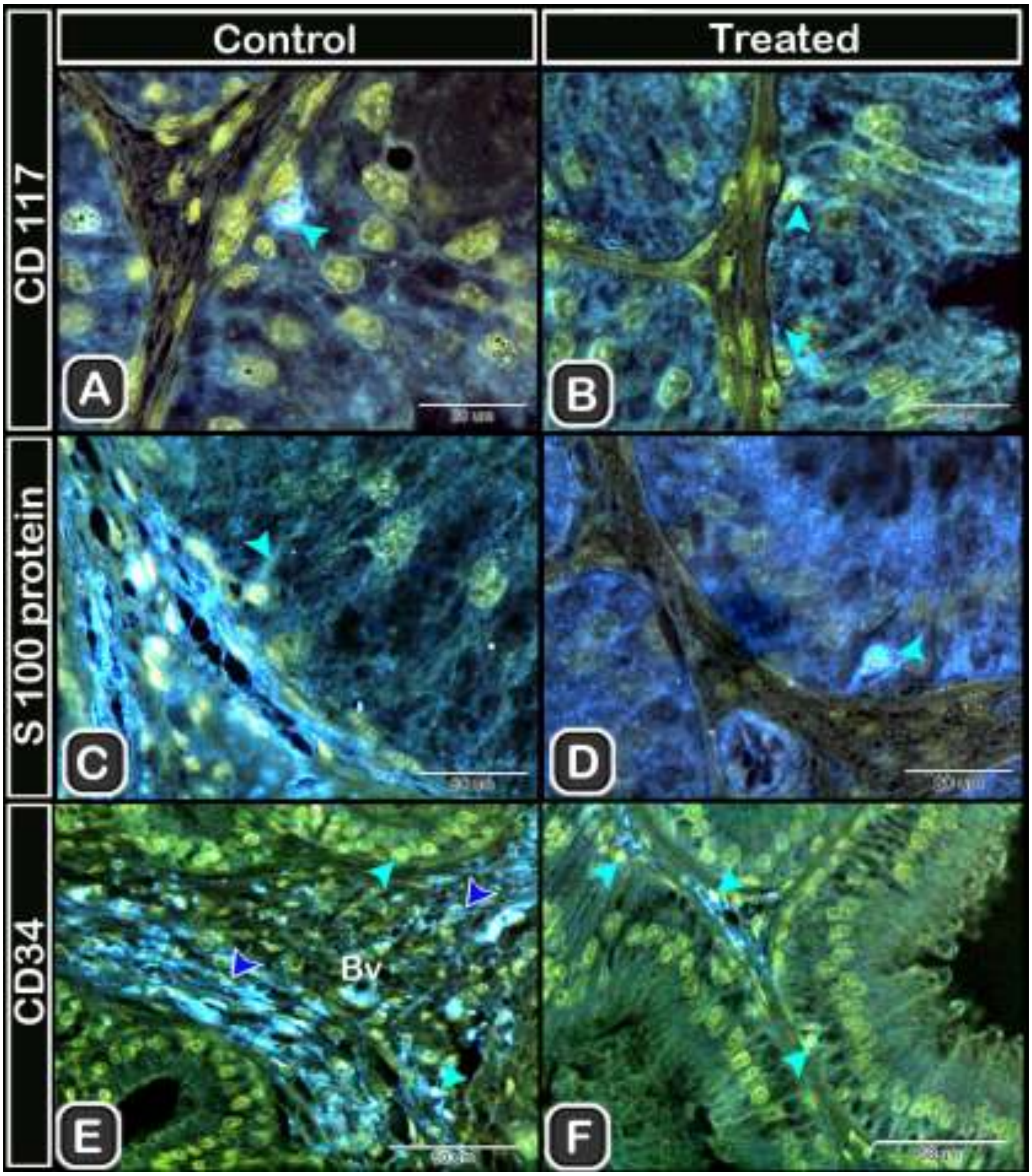

Supplementary legend 4: Negative images for organization of Cd117, S100 protein and CD34 positive DCs (green arrowheads) and telocyted (blue arrowheads) in Soay ram seminal gland. (A, C, E) control groups (B, D, F) melatonin treated groups by using CMEIAS Color Segmentation.

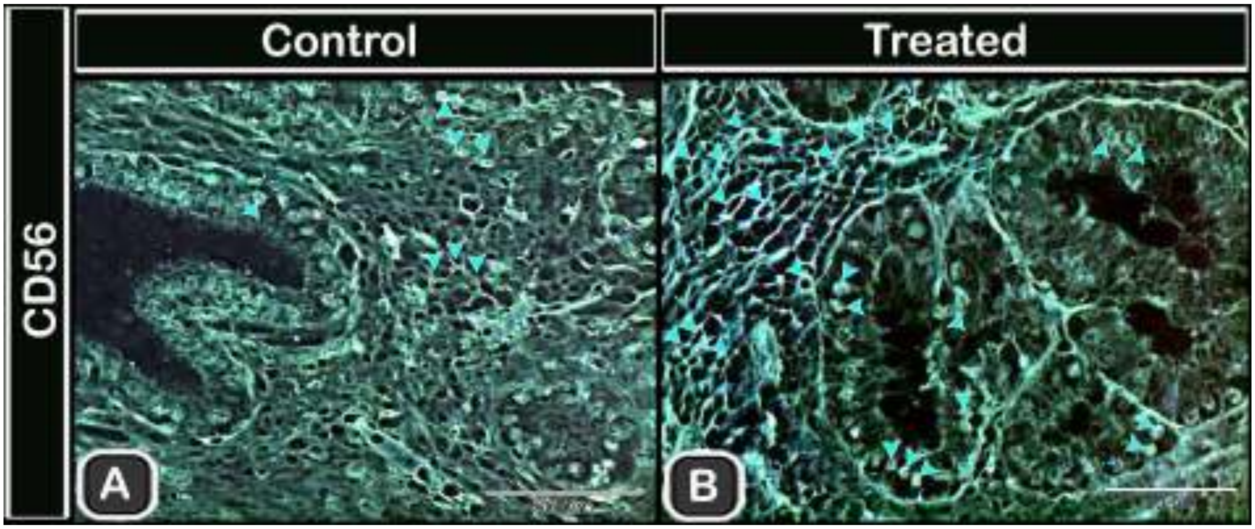

Supplementary legend 5: Negative images for organization of CD56 positive DCs (green arrowheads) in Soay ram seminal gland. (A) control groups (B) melatonin treated groups by using CMEIAS Color Segmentation.

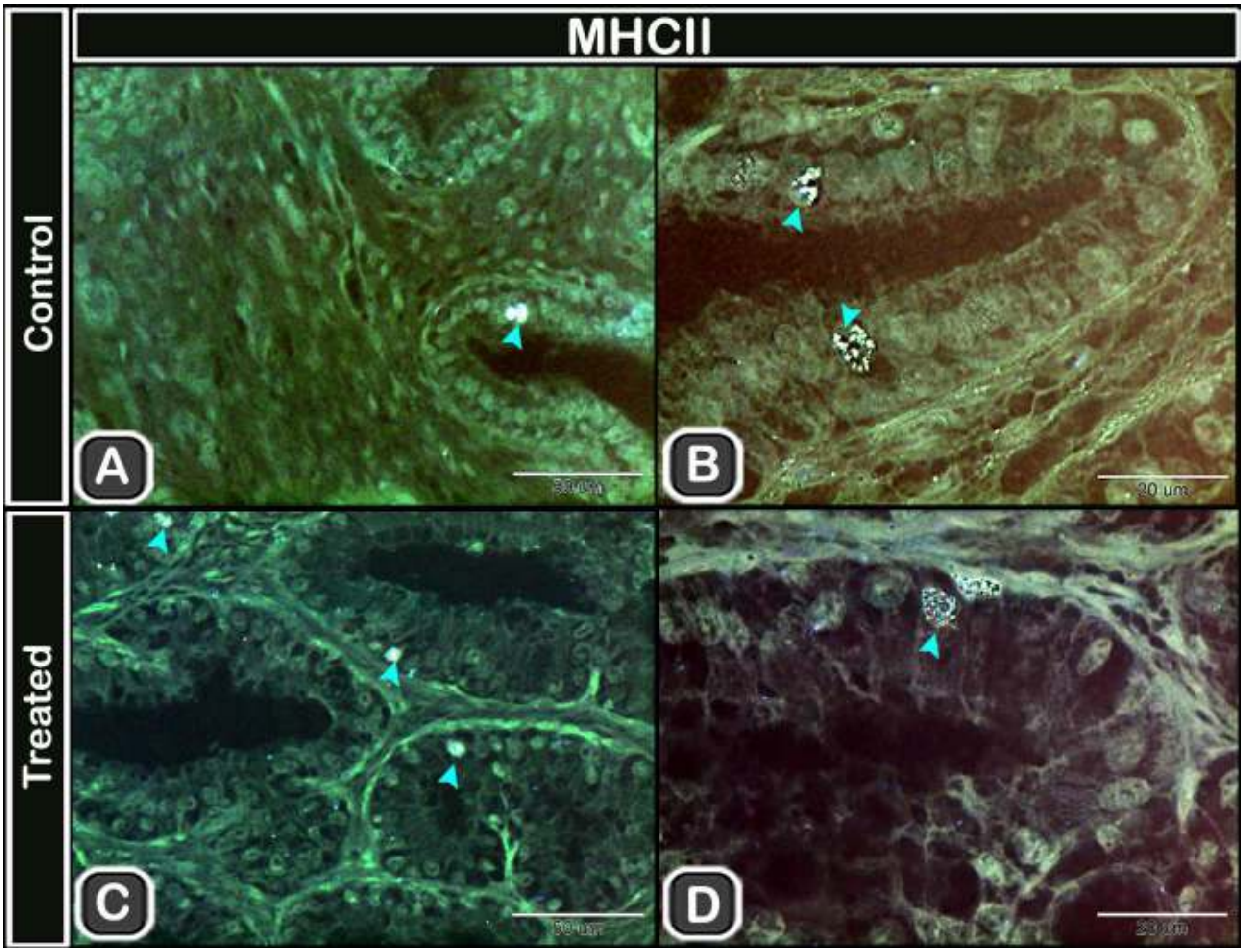

Supplementary legend 6: Negative images for organization of MHCII positive DCs (green arrowheads) in Soay ram seminal gland. (A, B) control groups (C, D) melatonin treated groups by using CMEIAS Color Segmentation.

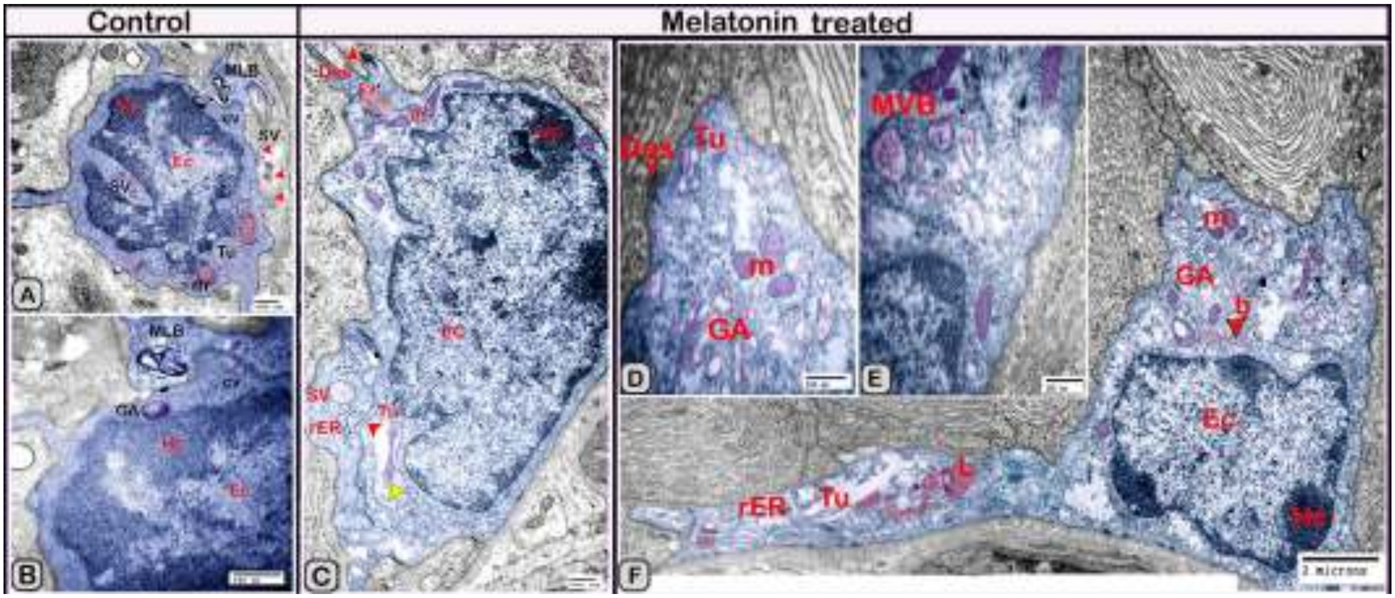

Supplementary legend 7: abstract figure of digitally coloured transmission electron microscope images and negative showing a stimulatory effect of melatonin on the DCs, which was associated with increment in the secretory activity of DCs. A and B the dendritic cells in the control groups had a deeply indented nucleus and a great amount of the peripheral heterochromatin. The cytoplasm contained few cell organelles. C, D, E, F the dendritic cells in the melatonin treated groups with abundant cell organelles and euchroamtic nucleus. The cytoplasmic cell processes were thin and short. Abundant mitochondria and well-developed Golgi apparatus, Birbeck granules, multilamellar bodies were observed. The endosomal lysosomal system was more developed compared to the control group.

Abbreviation: heterochromatin (HC), Euchromatin (EC), mitochondria (m), Golgi apparatus (GA), cavolae (cv), secretory vesicles (SV, arrowhead). multilamellar bodies (MLB), tubules (Tu), Birbeck granules (b, arrowhead), multivesicular bodies (MVB), phagolysosomes (L)

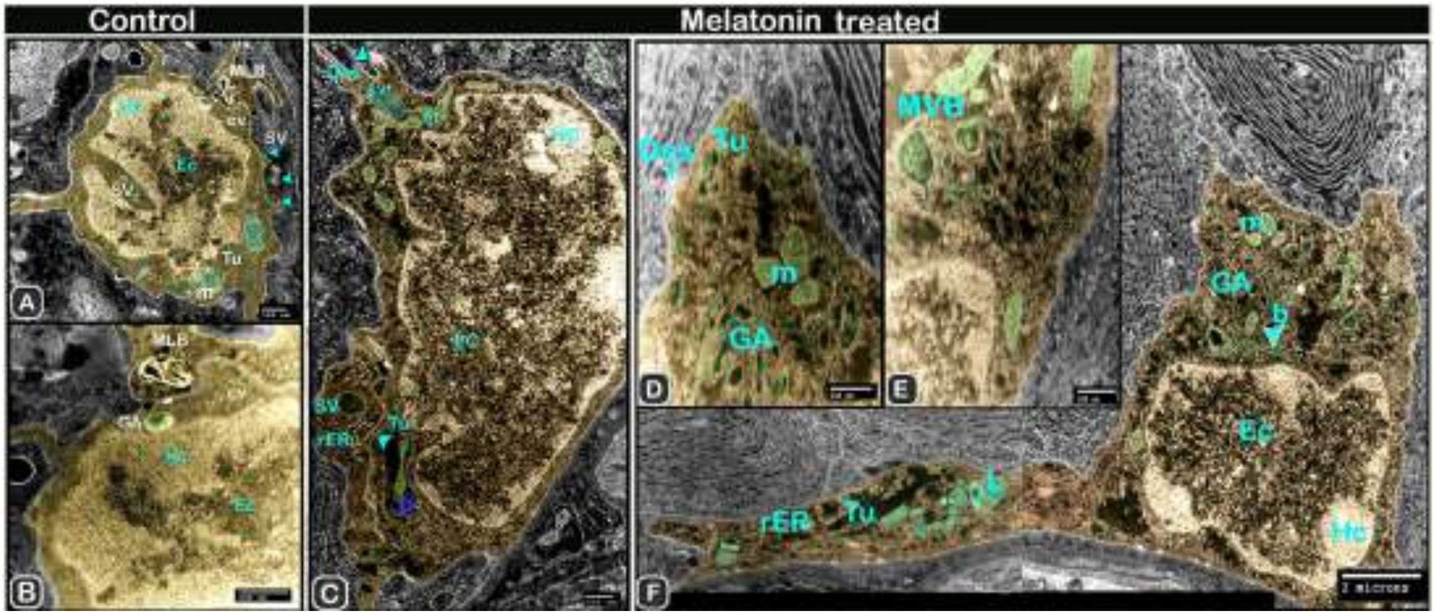

Supplementary legend 8: Negative images by using CMEIAS Color Segmentation showing a stimulatory effect of melatonin on the DCs, which was associated with increment in the secretory activity of DCs. A and B the dendritic cells in the control groups had a deeply indented nucleus and a great amount of the peripheral heterochromatin. The cytoplasm contained few cell organelles. C, D, E, F the dendritic cells in the melatonin treated groups with abundant cell organelles and euchroamtic nucleus. The cytoplasmic cell processes were thin and short. Abundant mitochondria and well-developed Golgi apparatus, Birbeck granules, multilamellar bodies were observed. The endosomal lysosomal system was more developed compared to the control group. Abbreviation: heterochromatin (HC), Euchromatin (EC), mitochondria (m), Golgi apparatus (GA), cavolae (cv), secretory vesicles (SV, arrowhead). multilamellar bodies (MLB), tubules (Tu), Birbeck granules (b, arrowhead), multivesicular bodies (MVB), phagolysosomes (L)
